# Protocol for the YORKSURe prospective multistage study testing the feasibility for early detection of bladder cancer in populations with high disease-specific mortality risk

**DOI:** 10.1136/bmjopen-2023-076612

**Published:** 2023-09-07

**Authors:** James WF Catto, Bernard North, Megan Goff, Abigail Carter, Michelle Sleeth, Olena Mandrik, Jim Chilcott, Peter Sasieni, Marcus G K Cumberbatch

**Affiliations:** 1 Department of Urology, Sheffield Teaching Hospitals NHS Trust, Sheffield, UK; 2 Division of Clinical Medicine, University of Sheffield, Sheffield, UK; 3 Cancer Prevention Trials Unit, King's College London, London, UK; 4 School of Health and Related Research, The University of Sheffield, Sheffield, UK; 5 Health Economics and Decision Science, University of Sheffield, Sheffield, UK

**Keywords:** bladder disorders, mass screening, urological tumours

## Abstract

**Introduction:**

Around 25% of patients with bladder cancer (BCa) present with invasive disease. Non-randomised studies of population-based screening have suggested reductions in BCa-specific mortality are possible through earlier detection. The low prevalence of lethal disease in the general population means screening is not cost-effective and there is no consensus on the best strategy. Yorkshire has some of the highest mortality rates from BCa in England. We aim to test whether population screening in a region of high mortality risk will lead to a downward stage-migration of aggressive BCa, improved survival and is cost-effective.

**Methods and analysis:**

YORKSURe is a tiered, randomised, multicohort study to test the feasibility of a large BCa screening randomised controlled trial. In three parallel cohorts, participants will self-test urine (at home) up to six times. Results are submitted via a mobile app or freephone. Those with a positive result will be invited for further investigation at community-based early detection clinics or within usual National Health Service (NHS) pathways. In Cohort 1, we will post self-testing kits to research engaged participants (n=2000) embedded within the Yorkshire Lung Screening Trial. In Cohort 2, we will post self-testing kits to 3000 invitees. Cohort 2 participants will be randomised between haematuria and glycosuria testing using a reveal/conceal design. In Cohort 3, we will post self-testing kits to 500 patients within the NHS pathway for investigation of haematuria. Our primary outcomes are rates of recruitment and randomisation, rates of positive test and acceptability of the design. The study is currently recruiting and scheduled to finish in June 2023.

**Ethics and dissemination:**

The study has received the following approvals: London Riverside Research Ethics Committee (22/LO/0018) and Health Research Authority Confidentiality Advisory Group (20/CAG/0009). Results will be made available to providers and researchers via publicly accessible scientific journals.

**Trial registration number:**

ISRCTN34273159.

**STRENGTHS AND LIMITATIONS OF THIS STUDY**

- The multicohort approach allows for contemporaneous assessment of each facet of the study while permitting the participant pathway to be dynamically optimised.
- The conceal/reveal design offers high compliance and low contamination, but may be concerning to participants.
- Organisational logistics to implement such a study design is exhaustive and relies on collaboration with several industry vendors.
- The acceptability and usability of the study platforms are uncertain.
- The sensitivity of a diagnostic pathway without initial flexible cystoscopy is unknown.

## Introduction

Bladder cancer (BCa) is a common malignancy and one of the most expensive to manage.^1 2^ BCas are best stratified into non-muscle-invasive (NMI) and muscle invasive (MI) cancers. In the UK, around 25% of patients present with MI BCa which requires radical treatment in an attempt at cure.^3 4^ BCa rates increase with age, smoking and occupational carcinogen exposure.^5–7^ Consequently, BCa incidence and mortality demographics often match local risk factor patterns of exposure. Yorkshire has some of the highest incidence and mortality rates of BCa in England, perhaps reflecting lifestyle choices and local industries.^2 8–10^

In contrast to many human malignancies, disease-specific survival for BCa is not improving,^2 11^ suggesting a need to change the approach to manage this cancer. Many BCa’s present late to healthcare professionals as the symptoms mirror those of common benign conditions, such as bacterial cystitis, detrusor overactivity or prostatic enlargement.^12^ Within the NHS, more BCas are diagnosed following routine General practitioner (GP) referral (41.3% (CI: 40.7% to 42.0%)) for urinary symptoms than via 2 week-wait (2ww) pathways (27.5% (CI: 26.9% to 28.1%)), and the rate of emergency presentation increases with advancing tumour stage, from 6.4% for NMI to 18.8% for MI BCas.^2^

### Current knowledge: BCa early detection

Most BCas are diagnosed in persons with visible haematuria (VH) or non-visible haematuria (NVH).^13^ As such, there is potential to use haematuria to detect asymptomatic disease earlier than would occur otherwise.^14^ Indeed, patient surveys reveal many individuals complain of irritative bladder symptoms (dysuria, frequency) prior to developing VH.^15 16^ However, there have been few attempts at screening for BCa using haematuria. Two non-randomised single arm studies found that haematuria testing in asymptomatic persons leads to earlier stage of cancers at detection and may improve survival.^17 18^ Britton *et al* screened 2356 British men and found 17 BCas, of which all were NMI.^19^ At 7 years, 33% of the high-grade NMI cohort had died from BCa, reflecting potential undermanagement of this aggressive cancer.^20^ Messing *et al* screened 1575 American men and found a lower stage at diagnosis, with reduced BCa mortality rate (0%) in screened men compared with rates in men from a matching state-wide registry (16.4% mortality rate).^18^ The authors concluded that screening did reduce mortality, but it needs to be applied in high-risk populations and that haematuria is intermittent in patients with BCa.^21^ In populations exposed to aristolochic acid, Zlotta *et al* found survival (0% mortality) occurred only with screening (100% mortality in non-screened patients),^22^ supporting the potential for BCa screening to be cost-effective in high-risk populations.^23^

### Research need

YORKSURe is a feasibility trial aimed at the early diagnosis of BCa through the detection of haematuria in asymptomatic individuals at high risk of mortality from the cancer. Following results of the feasibility trial, the aim is to conduct a large-scale trial, with sufficient power to test any differences in BCa mortality. However, to undertake this successfully, we need to understand if our proposed approach is robust, appropriate, necessary and acceptable to participants. Here we detail the trail using Protocol V.5.0 date 16 January 2023.

#### Study aims and objectives

Three cohorts will be studied in this feasibility study. Each will address a separate aim, all of which are needed to develop a phase III prospective trial:

- Aim 1: Cohort 1 will test compliance, prevalence of haematuria and BCa and the economics of the trial within a screening-engaged population. It might suggest that BCa screening works best when embedded within lung screening services. It will also test women and populations younger than typical for the highest risk BCa age group.
- Aim 2: Cohort 2 will test compliance, prevalence of haematuria and BCa, prevalence of glycosuria and undiagnosed diabetes within a high-risk community-based population.
- Aim 3: Cohort 3 will provide a positive predictive value (PPV) of the study tests (urine self-testing, urine cytology and ultrasound) as assessed in a population already referred in the NHS 2ww haematuria pathway.

## Methods and analysis

### Study design and setting

**Figure 1:**
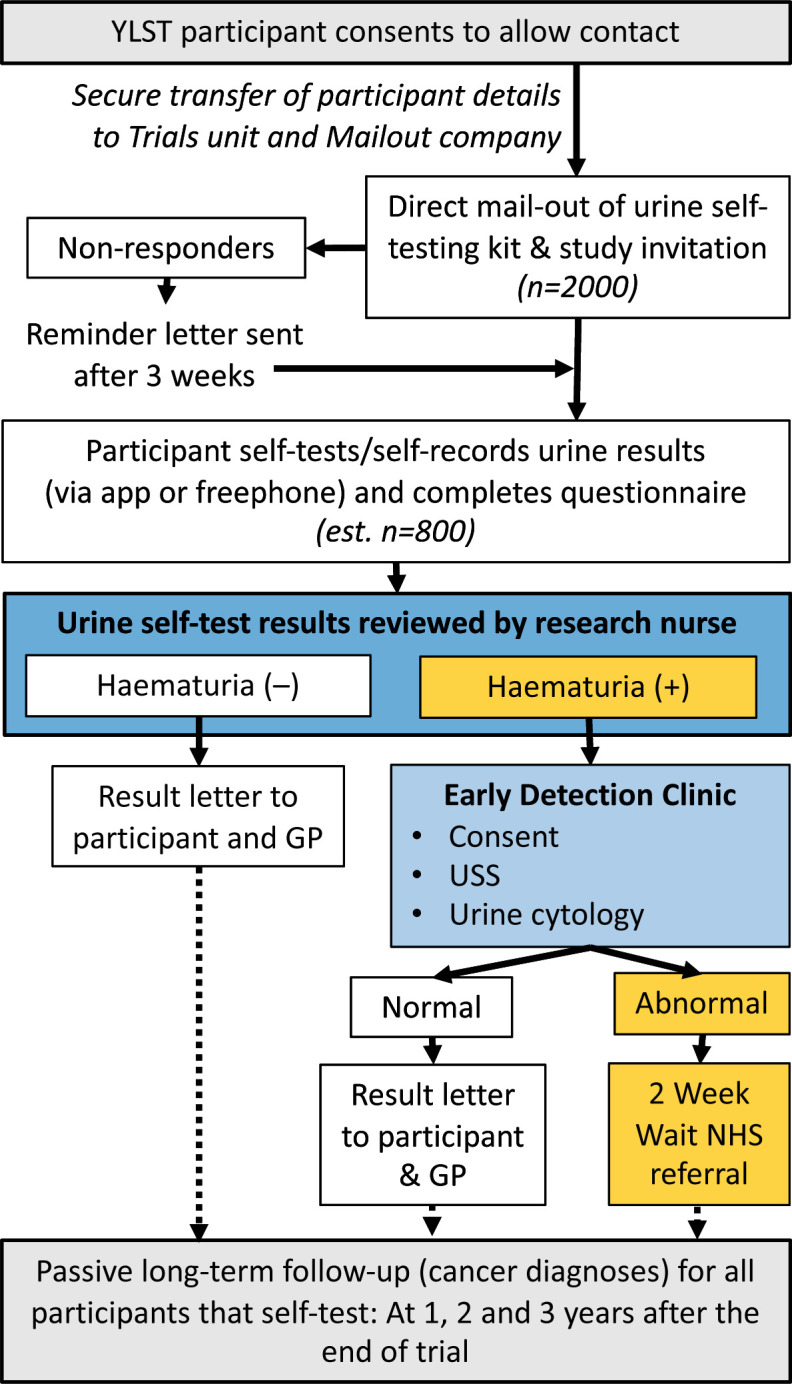
Recruitment and flow within Cohort 1 within the YORKSURe study: male and female participants will be solely recruited from the YLST. USS, ultrasound scan; YLST, Yorkshire Lung Screening Trial.

Eligibility for Cohort 1: men and women aged 55–80 years who are registered with a GP within the Leeds Clinical Commissioning Group area, who are part of the Yorkshire Lung Screening Trial^24^ and have given consent to be contacted by other research teams (figure 1).

**Figure 2:**
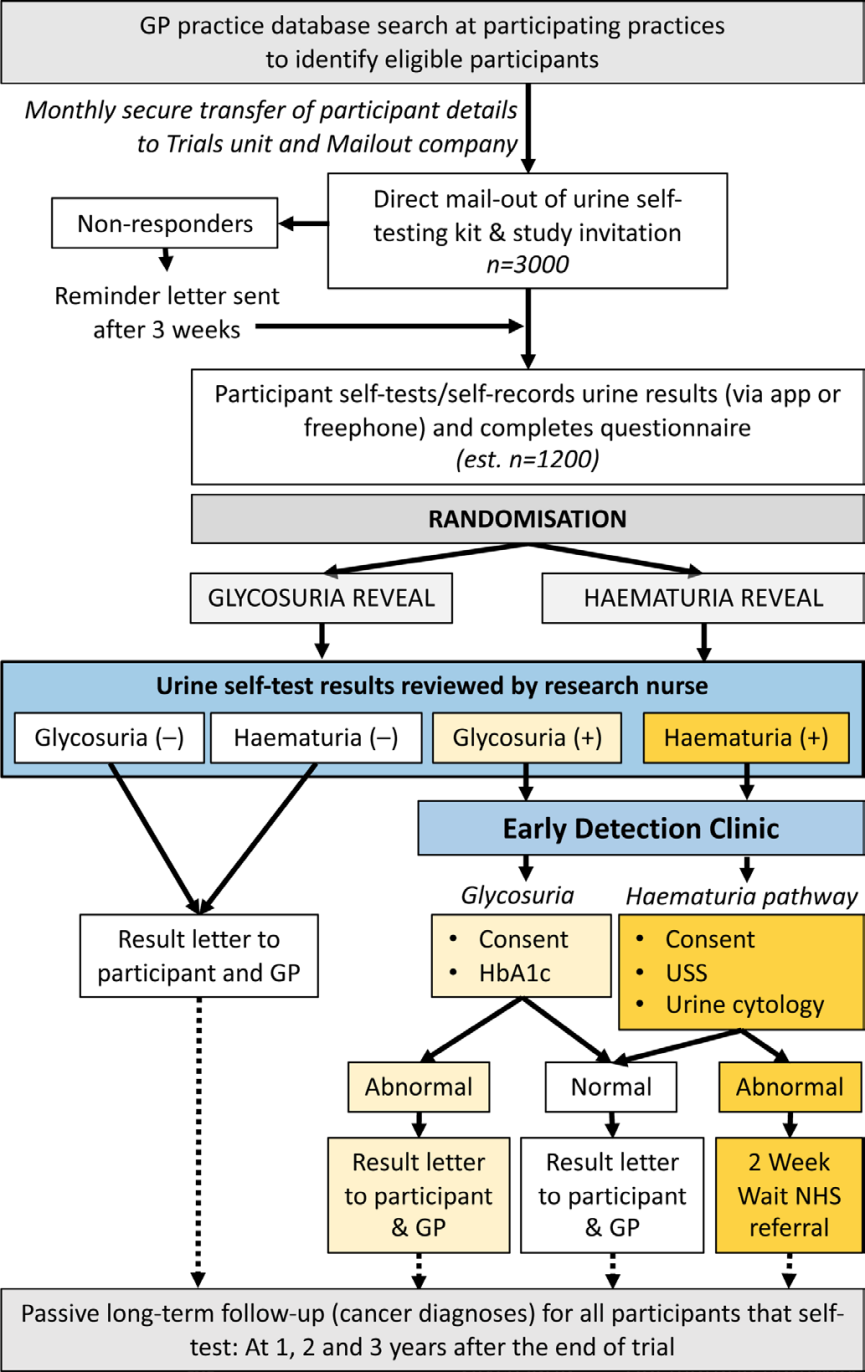
Recruitment and flow within Cohort 2 of the YORKSURe study: male participants will be invited from populations at risk of high-mortality from bladder cancer. USS, ultrasound scan.

Eligibility for Cohort 2: Men aged 65–79 identified through a database search at 8–10 selected GP practices considered to be in high-risk BCa mortality areas in South Yorkshire (figure 2).

**Figure 3:**
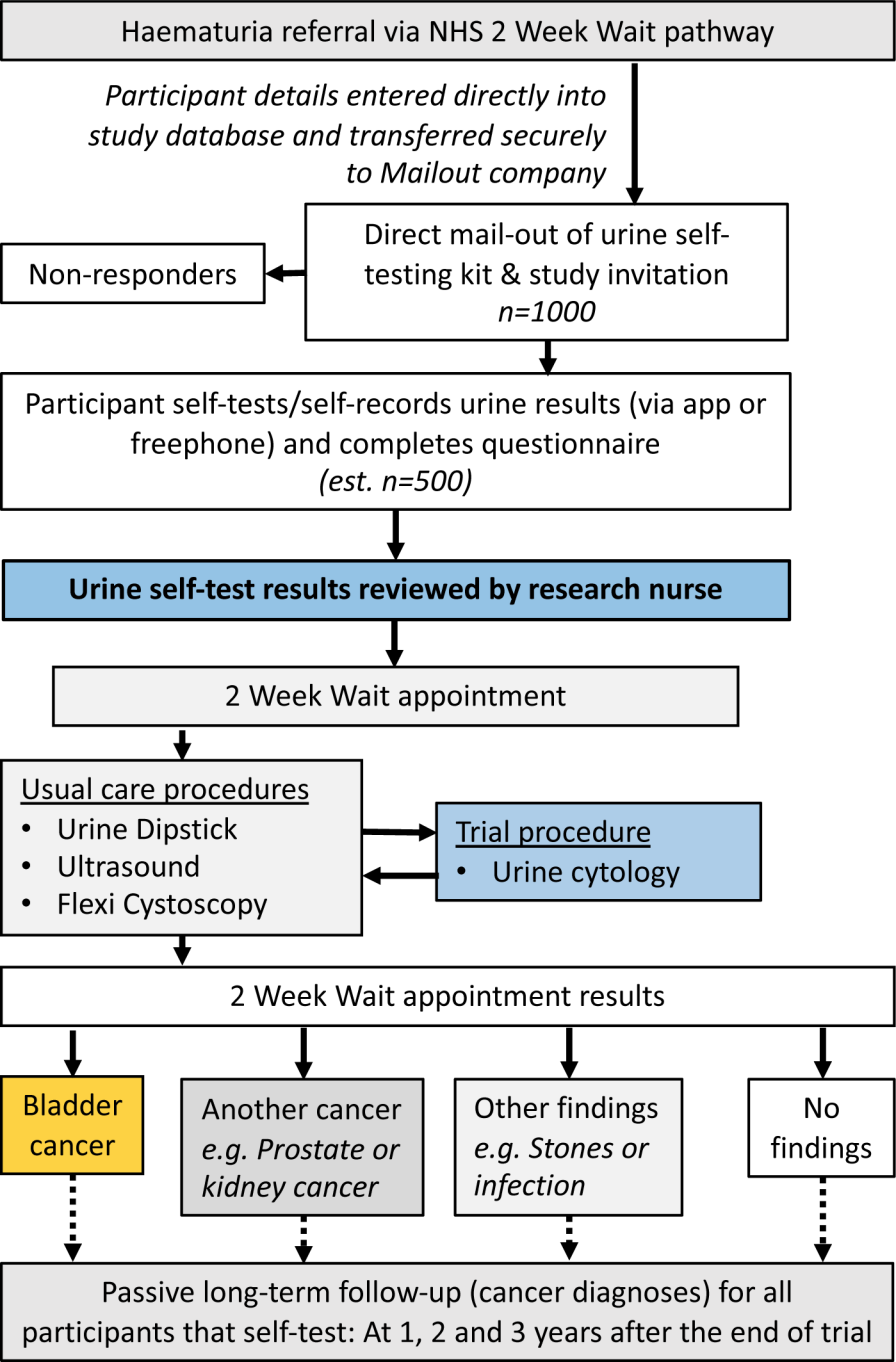
Participant flow within Cohort 3 of the YORKSURe study: male and female patients undergoing investigation within the NHS for haematuria (according to the 2 week-wait criteria within the National Institute of Care Excellence guidelines) will be invited to participate.

Eligibility for Cohort 3: men and woman will be identified via local urology departments when a referral is made for a 2ww urology appointment for investigation of haematuria (figure 3).

### Exclusion criteria

#### Cohort 1

1. Unable to or did not consent to contact from other research teams.
2. Insufficient capacity to give consent to take part in the research as determined by the investigator taking consent.

#### Cohort 2

1. Participant has opted out of their NHS data being used for research via the National Data Opt-Out.
2. Prior diagnosis of bladder or kidney cancer.
3. Diagnosis with any other cancer within the past 5 years (except non-melanoma skin cancer).
4. Insufficient capacity to give consent to take part in the research, defined as the presence of the following conditions coded in the GP records: dementia, Alzheimer’s disease or Parkinson’s disease; or as determined by the investigator taking consent.

#### Cohort 3

1. Unable to provide written informed consent.
2. Insufficient capacity to give consent to take part in the research, defined as the presence of the following conditions coded in the GP records: dementia, Alzheimer’s disease, or Parkinson’s disease; or as determined by the investigator taking consent.
3. Evidence in patient medical record of historic dissent to use of data in research or participant has opted out of data use via the National Data Opt-Out.

### Screening tests and self-testing

Participants in all three cohorts will be provided with home urine self-testing kits produced and supplied by Testcard. The kit contains 6× Roche Combur 5 HC test strips for the graduated determination of glucose, leucocytes, nitrites, protein and erythrocytes. Blood and glucose will be tested for primary study purposes. The other three parameters (leukocytes, nitrite, protein) will be analysed as part of an exploratory objective.

Participants will record six continuous days of test strip results via a bespoke study App or study freephone service. Prior to commencing self-testing, participants will complete a bladder symptom questionnaire (a modified and unvalidated version of the Urinary Tract Infection Symptom Assessment (UTISA) Questionnaire).^25^ The questionnaire will collect demographic data and information on bladder symptoms within the past 4 weeks including presence of VH. The questionnaire will be pseudonymised and all data pertaining to the questionnaire will be kept separate from urine self-testing results until the end of the trial. The app will capture a digital photograph of the test strip, which will be interpreted by a research nurse against the Roche Combur 5 HC colour chart. Test strip results will not be available to participants at the point of self-testing.

It is anticipated and expected that some participants in the study population will either not have a smartphone, or will not be confident in using a mobile App. A study freephone service will therefore be available as an alternative option for recording self-test results. The freephone service will be an automated system whereby participants use their keypad to enter their responses to the questionnaire and test strip results. The freephone service requires confirmation of the test strip colour result by the participant against the Roche Combur 5 HC colour chart, whereas App users will simply take a photograph of their completed test strip which will be submitted for interpretation by a research nurse.

### Investigation of haematuria

Participants in Cohort 1 and those randomised to the haematuria arm of Cohort 2 will be investigated for the presence of haematuria (figures 1 and 2). If a single positive result is identified on research nurse review of the erythrocyte test parameter, then the participant will be invited to attend an Early Detection Clinic (run in a nearby GP surgery and staffed by study-employed or NIHR Clinical Research Network (CRN)-employed nursing staff) within 4–6 weeks of assessment of their results. At this clinic, participants will undergo a urinary tract ultrasound scan (USS) and provide a urine sample for cytology. Those with an abnormal USS (bladder or renal mass suggestive of cancer detected) or abnormal cytology will be referred by their GP or the research nurse team (following standard NHS 2ww criteria) to their local urology haematuria service for evaluation (flexible cystoscopy and additional assessments). We expect that around 20% of participants with self-testing haematuria will require referral. The test results for these participants will be obtained directly through the relevant urology departments and categorised into: BCa diagnosis, kidney cancer diagnosis, upper tract urothelial cancer diagnosis, urinary stones, infection or inflammation, stricture, clinically benign prostatic enlargement, post-TURP (transurethral resection of the prostate), diagnosis unclear/further tests planned, likely prostate cancer, no abnormal finding or other finding. Participants with normal USS and normal cytology, from the early detection clinic, will receive a letter explaining this and discharging them from the study. They will receive advice about the need to see their GP if they have urinary symptoms or VH. Their GP will receive a letter with advice about management of NVH. Participants without haematuria will receive a letter confirming their result and informed that their GP will also be notified of their results. They will be asked to seek a GP appointment if they develop urinary symptoms in the future. Participants in cohort 3 will undergo standard NHS assessment of haematuria as part of their referral process (regardless of their test strip results). This includes cystoscopy and upper tract imaging (typically CT urogram and USS).

### Investigation of glycosuria

Participants randomised to the glycosuria arm of Cohort 2 will be investigated for the presence of glycosuria. Glycosuria was chosen as it is a validated urine test that can detect a meaningful disease (diabetes mellitus), whose outcomes are better detected at an early stage. If a single positive result is identified on research nurse review of the glucose test parameter, then the participant will be invited to attend an Early Detection Clinic within 4–6 weeks of assessment of their results. At the clinic, participants will undergo hemoglobin A1c (HbA1c) blood testing. Those with elevated HbA1c (as defined by local reference ranges) will be referred to their GP for diabetic management. Those with a normal HbA1c (as defined by local reference ranges) will receive a letter explaining that their test was normal and will be advised to see their GP if they think they have diabetic symptoms. Their GP will also be informed of the test result. Participants in the glucosuria testing arm without glycosuria will receive a letter confirming their normal result and informed that their GP will also be notified of their results. Participants will be advised to seek a GP appointment if they develop diabetic symptoms.

### Consent

On receipt of the postal urine self-testing kit, participants will be able to choose an action to take:

1. Consent to enter trial: those wishing to take part in the trial will be asked to consent to participate by agreeing to consent statements in the mobile App or freephone system, before proceeding to complete the study questionnaire, and then self-testing with the test strips. Invitees will be prompted prior to providing results to have read the Participant Information Sheet.
2. Non-response: non-responders (ie, do not consent or provide test results via the mobile App or freephone)

Following a positive urine self-test result for haematuria (Cohort 1) and haematuria/glucosuria (Cohort 2) participants will receive a telephone call from a study research nurse to explain their results and schedule an Early Detection Clinic appointment.

This will be followed by the mailout of a second Patient information sheet (PIS) detailing what will happen at the Early Detection Clinic appointment. At the clinic, written informed consent will be obtained prior to any trial-specific procedures (online supplemental material). The chief investigator (CI) will ensure that those with delegated responsibility to obtain informed consent are duly authorised, trained and competent according to the ethically approved protocol, principles of Good Clinical Practice (GCP) and Declaration of Helsinki. The right of a participant to refuse participation without giving reasons will be respected.

10.1136/bmjopen-2023-076612.supp1 Supplementary data

Cohort 3 participants will receive a self-testing kit including a study invitation letter and PIS detailing what will happen at the 2ww appointment (figure 3). The invitation letter will be from the routine care team at the participating urology department. On presentation to the 2ww appointment, written informed consent will be obtained prior to any trial-specific procedures. Translation via hospital interpreters is acceptable if required. The principal investigator will ensure that those with delegated responsibility to obtain informed consent are duly authorised, trained and competent according to the ethically approved protocol, principles of GCP and Declaration of Helsinki. The right of a participant to refuse participation without giving reasons will be respected. For Cohort 3, participation is mostly altruistic as the participants will already be in the NHS pathway and will have the gold-standard tests for detection of BCa.

### Sample size(s)

A formal power calculation is not appropriate for this trial due to its feasibility-related outcomes. The current planned sample size is to invite 2000 participants in Cohort 1, (estimated 800 enrolled), invite 3000 male participants in Cohort 2, (estimated to enrol and randomise 1200) and invite 1000 participants in Cohort 3 (estimated enrolment of 500).

### Randomisation

Cohort 2 will be randomised between two arms where either the haematuria or glycosuria result will be revealed during the trial. Participants will be randomised via permuted blocks to ensure approximately equal numbers in the two arms. The randomisation lists will be generated by the trial statistician. The central nursing team will randomise participants prior to review of self-test results. Participants will not be given any details of the allocated group until they receive their initial results letter.

### Primary outcomes

This is a feasibility study and so there are multiple primary outcomes reflecting each stage of the study and what we need to understand for a larger phase III randomised controlled trial:

1. Acceptability of urine self-testing and the Early Detection Clinics will be measured by:

1.1. Proportion of invitees who report results for at least one test strip.

1.2. Proportion of participants who attend and consent for cytology and urinary tract ultrasound following detection of haematuria on self-testing.

1.3. Proportion of participants who attend and consent for HbA1c blood testing following the detection of glycosuria on self-testing.

2. Compliance with urine self-testing will be measured by;

2.1. The proportion of participants who report results for (i) all six test strips and (ii) three or more test strips.

2.2. The number of test strips returned on average in those who return at least one test strip.

3. Prevalence of haematuria will be measured from the proportions of participants with ≥1 test strip positive for haematuria.

4. Prevalence of glycosuria will be measured from the proportions of participants with ≥1 test strip positive for glycosuria.

5. Haematuria clinic outcomes will be measured via:

5.1. The proportion of participants with a positive or abnormal cytology in those where a urine sample is collected.

5.2. The proportion of participants who have an abnormal USS, who underwent scanning.

6. Glycosuria clinic outcomes will be measured via the proportions of those with an elevated HbA1c following collection of a blood sample.

7. Urinary symptoms will be measured via symptom scores from the urine symptoms questionnaire (as completed prior to self-testing).

8. BCa in the tested population will be measured via the proportions of BCa cases and corresponding stage in identified participants (excluding non-responders and withdrawals).

9. The accuracy of self-testing, cytology and USS will be measured via:

9.1. The sensitivity, specificity, PPV and negative predictive value (NPV) of the following in men and women with cancer as identified by cystoscopy results at the 2ww appointment

9.1.1. Self-testing (App vs freephone).

9.1.2. Cytology and ultrasound (separately and in combination) as a triage test in those with positive haematuria

9.2. The predictive value of symptoms in combination with self-testing:

9.2.1. Haematuria positive/symptoms positive

9.2.2. Haematuria negative/symptoms positive

9.2.3. Haematuria positive/symptoms negative

9.2.4. Haematuria negative/symptoms negative

10. Recruitment rates will be measured via the number of invitations sent out versus uptake.

### Secondary and exploratory outcomes

Secondary outcomes include (1) self-test reporting via the mobile app or freephone, and this impact on sensitivity/specificity to detection of cancer, and (2) the acceptability of the reciprocal design (measured by the proportions of compliance/attendance/surveys at the end of the trial).

Exploratory outcomes include (1) Comparison of populations, demographics and cancer incidence in those who do/do not complete self-testing (comparing the characteristics where available including sex, age group, quintile of deprivation, smoking history). All participants who self-test will be flagged in National Cancer Registration and Analysis Service (NCRAS) for cancer diagnoses at 1, 2 and 3 years following the end of the trial. (2) The development of an artificial intelligence (AI) platform for the assessment of urine samples for cytology analysis for the future study will be measured by the accuracy of the AI read versus the manual read at the end of the trial. (3) The potential harm of cystoscopy adverse effects, specifically urinary tract infections requiring medical treatment, will be measured by the proportions of participants who have a Urinary Tract Infection (UTI) requiring medical treatment following cystoscopy at the end of the trial. (4) Development of a health economics model^23^ using the information from this feasibility study that could be used to develop a future community-based screening programme for BCa will be measured at the end of the study by: (4.1) Resource usage in each arm (number of clinics used, blood tests, ultrasound tests, extra NHS tests); (4.2) Rate of cancers and other illnesses per arm. (5) The addition of other urinary contents (leucocytes, nitrate, protein) in relation to the risk prediction of BCa will be measured by other positive test strip panels in addition to haematuria at the end of the trial.

### Data collection

Self-test results and questionnaire data will be captured in the bespoke mobile app and freephone system. The questionnaire will collect information on diabetes (diagnosis and management), smoking status, ethnic group, lesbian, gay, bisexual, and transgender (LGBT) identification, highest level of education, history of factory work as well as bladder symptoms (via a modified and unvalidated version of the UTISA Questionnaire^25^). Cohort 1 and 2 data collected at Early Detection Clinics will be entered into a REDCap^26^ database and will include height and weight, current occupation, previous longest occupation, concomitant medication, ultrasound results, urine cytology results and HbA1c results. Data for Cohort 1 and 2 participants who are subsequently referred for a 2ww, and consenting Cohort 3 participants, will be obtained via medical records to include results of cystoscopy, ultrasound/CT, cytology and cancer staging where applicable. At 14–30 days post cystoscopy, these participants will be contacted to collect information of any treated incidences of UTI following cystoscopy. Passive follow-up data (identification of cancer diagnosis and stage) will be collected through the NCRAS at 1, 2 and 3 years after the end of the trial for all participants who have self-tested (excluding withdrawals). Summary data will also be collected through NCRAS, requested by geographic area, for a population comparable to Cohort 2.

### Statistical analysis

We will present estimates of percentages, with 95% Wilson CIs, on a per cohort basis for the three trial endpoints that will allow assessment of feasibility and inform the subsequent more definitive trial. We will estimate acceptability (proportion of invitees consenting and returning a test strip), compliance (proportion of participants returning at least three or all six test strips), haematuria and glucosuria, proportion of positive outcomes for those referred to the Early Detection Clinic (positive cytology and/or USS for haematuria and HbA1c for glycosuria). We will assess the levels of BCa in the cohort identified by Early Detection clinic and at 3 years using National Disease Registration Service (NDRS, formerly NCRAS) and the accuracy of the haematuria test results including PPVs and NPVs for Cohorts 1 and 2 only. The urinary symptoms questionnaires will be summarised by cohort, by the proportions with each urinary symptom, and by overall urinary symptoms using mean, SD, median, minimum, maximum and quartiles. Missing values will be imputed as no symptom.

### Economic evaluation

An early health economic model of haematuria screening for BCa in high-risk areas will be developed, including epidemiology, natural history and how this is affected by screening.^23^ The model design will be informed through analysis of other screening and diagnostic models in BCa. The model will be parameterised with the generated feasibility outcome data, supplemented by evidence from published literature. Bayesian calibration methods will be used for parameterisation of the disease natural history and screening model. The model will input to the design of and be refined using the subsequent trial outcomes.

### Patient and public involvement (PPI)

A group of nine PPI representatives were approached for initial review of the trial design, objectives, lay summary and survey questions, and their responses have been incorporated in the trial design. PPI members have also reviewed the participant facing documentation and provided feedback. One-to-one interviews and discussions with PPI members were held to obtain specific feedback on aspects of how the study is presented to potential participants. A PPI lead is included in the Trial Steering Committee (TSC).

### Trial organisation and administration

The trial is sponsored by the University of Sheffield and coordinated by the Cancer Research UK & King’s College London Cancer Prevention Trials Unit (CPTU). The central research nursing team and central cytology laboratory is based at Sheffield Teaching Hospital NHS Foundation Trust.

Two trial oversight groups provide guidance and management for the three cohorts. The Trial Management Group meet at least monthly and will comprise the CI, representatives from University of Sheffield and CPTU who are responsible for day-to-day management of the trial and carrying out the work detailed in the trial protocol. The purpose of the group is to ensure the trial is on track to its project plan and budget, manage trial risks, issues and mitigations and to escalate and report any major risks and issues to the TSC as necessary.

The TSC will concentrate on progress of the trial, adherence to the protocol, participant welfare and to assess the impact and relevance of any new information of relevance to the research question. The TSC will make recommendations, through its Chair, to the TMG, CI, trial sponsor, trial funders, CPTU and any other relevant party on all appropriate aspects of the trial. Most members of the TSC, including the Chair, will be independent of the trial, and will include lay/patient representation. Non-independent members will also be part of the TSC as observers. The final decision regarding whether the trial may continue is the responsibility of the TSC.

### Ethics and dissemination

A risk assessment and review of ethical considerations has been performed as part of the trial, including development of mitigation strategies to ensure the safe and successful delivery of the trial. Key risks and mitigations from this trial have been outlined below:

1. Cancer screening is in part controversial due to the risks of finding and over treating indolent cancers. However, for BCa there is minimal morbidity in diagnosis (ie, from the transurethral resection of bladder tumour (TURBT)) and risk stratification is embedded in the treatment pathways. Many of these cancers are managed without radical treatment, and therefore there are few long-term side effects. Furthermore, autopsy studies^9^ reveal few undiagnosed BCa’s, suggesting that most tumours present clinically. Modelling of the disease natural history will aim to estimate overdiagnosis and will provide input to subsequent trial design to provide definitive evidence on these issues.
2. Another risk is that participants may attempt to seek the same test outside of the trial or try to identify what the colour change on their urinary test strip represents. We have worked with PPI representatives to tailor trial communications. It is key that participants understand that participation in the trial should be seen as altruistic and there is currently no evidence to suggest that screening of this may be beneficial and indeed may cause harm. Liaison with local GPs throughout the pilot period will be key in determining if they observe any increase in men or women requesting urinalysis assessments.
3. Variable practices in secondary care for the treatment of BCa (ie, post study) is another risk. Treating clinicians may use a wide variety of different regimens) to manage screen detected cancers. As part of the planned future trial, we will apply for funding for an embedded cohort study of comparative effectiveness and health-related quality of life (HRQOL) for these screen-detected cancers. Patients/clinicians will be able to choose from a selection of standard National Institute of Care Excellence approved pathways. HRQOL and cost will be collected within these pathways.

To ensure widespread dissemination, we plan to circulate the trial results to multiple research outputs including but not limited to conference proceedings and peer-reviewed journal articles relating to the trial design, results of the pilot trial, final uptake of participants and a final main analysis of primary and secondary endpoints encompassing economic modelling aspects. All trials will be published in high-impact journals, accompanied by a press release. This will be covered in more detail in the trial publication policy. Dissemination of trial results will be designed and delivered after extensive collaboration with PPI groups.

### Trial status

The trial opened to recruitment on 3 October 2022 and is in the active recruitment phase.

## Supplementary Material

Reviewer comments

Author's manuscript
