# Supplementary material for: Protocol for the YORKSURe prospective multistage study testing the feasibility for early detection of bladder cancer in populations with high disease-specific mortality risk
Source: BMJ Open. 2023 Sep 7;13(9):e076612. doi: 10.1136/bmjopen-2023-076612 (PMC10496676; doi:10.1136/bmjopen-2023-076612)
Supplement: Supplementary data [file bmjopen-2023-076612supp001.pdf]

Title: Yorkshire Screening of Urine trial (YORKSURe)

IRAS ID: 302276

Study ID:

INFORMED CONSENT FORM

Name of Researcher: Professor James Catto

		Please initial box
1	I confirm that I have read and understood the participant information sheet (dated...................version...........) for the above study. I have had the opportunity to consider the information, ask questions and have had these answered to my satisfaction.	☐
2	I understand that taking part is voluntary and that I am free to withdraw at any time without giving any reason, and without my medical care or legal rights being affected. I understand that samples and data collected prior to my withdrawal will continue to be used for the purposes of the study.	☐
3	I understand that relevant sections of my medical notes and data collected during the study, may be looked at by individuals from King's College London, University of Sheffield, or from regulatory authorities or from the NHS Trust, where it is relevant to my taking part in this research. Access to data by the organisations above may be given for monitoring and/or audit of the study to ensure that the research is complying with applicable research regulations. I give permission for these individuals to have access to my records and data.	☐
4	I understand that my personal data will be shared solely between members of the research team and participating study sites as part of the data analysis required for this study. As detailed in the participant information sheet, I am aware that employees of third-party providers contracted by King's College London may require access to my data to fulfil their role as a third-party service provider, but my records and information will be kept strictly confidential.	☐

Catto JWF, *et al. BMJ Open* 2023; 13:e076612. doi: 10.1136/bmjopen-2023-076612

**5**	I understand that the information collected about me will be used to support other research in the future and may be shared anonymously with other researchers or organisations which may include those in the commercial sector, here or abroad.	☐
**6**	**Applicable if providing a urine sample only:**<br>I understand that my anonymised urine sample (slide) may be used in future research, here or abroad, which has ethics approval. I understand this research may involve commercial organisations.	☐ Yes ☐ N/A
**7**	I agree to my General Practitioner being informed that I am taking part in the study and that my test results will be shared with them.	☐
**8**	I agree to take part in the above study.	☐

Name of participant	Date	Signature
Name of Person seeking consent	Date	Signature

Catto JWF, *et al. BMJ Open* 2023; 13:e076612. doi: 10.1136/bmjopen-2023-076612
