# Supplementary material for: Protocol for the YORKSURe prospective multistage study testing the feasibility for early detection of bladder cancer in populations with high disease-specific mortality risk
Source: BMJ Open. 2023 Sep 7;13(9):e076612. doi: 10.1136/bmjopen-2023-076612 (PMC10496676; doi:10.1136/bmjopen-2023-076612)
Supplement: Author's manuscript [file bmjopen-2023-076612.draft_revisions.pdf]

BMJ Open is committed to open peer review. As part of this commitment we make the peer review history of every article we publish publicly available.

When an article is published we post the peer reviewers' comments and the authors' responses online. We also post the versions of the paper that were used during peer review. These are the versions that the peer review comments apply to.

The versions of the paper that follow are the versions that were submitted during the peer review process. They are not the versions of record or the final published versions. They should not be cited or distributed as the published version of this manuscript.

BMJ Open is an open access journal and the full, final, typeset and author-corrected version of record of the manuscript is available on our site with no access controls, subscription charges or pay-per-view fees (http://bmjopen.bmj.com).

If you have any questions on BMJ Open's open peer review process please email info.bmjopen@bmj.com

BMJ Open

The YORKSURe study for early detection of bladder cancer: Feasibility assessments for implementing a targeted study in populations with high disease specific mortality risk: study protocol

Journal:	*BMJ Open*
Manuscript ID	bmjopen-2023-076612
Article Type:	Protocol
Date Submitted by the Author:	12-Jun-2023
Complete List of Authors:	Catto, James; University of Sheffield Medical School, Oncology and Metabolism<br>North, Bernard; King's College London,<br>Goff, Megan; King's College London<br>Carter, Abigail; King's College London<br>Sleeth, Michelle; King's College London<br>Mandrik, Olena; The University of Sheffield,<br>Chilcott, Jim; University of Sheffield, Health Economics and Decision Science<br>Sasieni, Peter; King's College London<br>Cumberbatch, Marcus; The University of Sheffield, Academic Urology
Keywords:	Bladder disorders < UROLOGY, Mass Screening, Urological tumours < ONCOLOGY

SCHOLARONE™
Manuscripts

The YORKSURe study for early detection of bladder cancer: Feasibility assessments for implementing a targeted study in populations with high disease specific mortality risk: study protocol

James W.F. Catto [1,2], Bernard North [3], Megan Goff [3], Abigail Carter [3], Michelle Sleeth [3], Olena Mandrik [4], Jim Chilcott [4], Peter Sasieni [3*] and Marcus G Cumberbatch [1,2*]

* Shared senior authorship

James Catto [Orcid ID = orcid.org/0000-0003-2787-8828]

Peter Sasieni [Orcid ID = orcid.org/0000-0003-1509-8744]

Affiliations:

1. Academic Urology Unit, University of Sheffield, Sheffield, UK; Department of Urology, Sheffield Teaching Hospitals NHS Trust, Sheffield, UK.
2. Department of Urology, Sheffield Teaching Hospitals NHS Foundation Trust, Sheffield
3. Cancer Prevention Group, School of Cancer and Pharmaceutical Sciences, Faculty of Life Sciences and Medicine, King's College London, London, United Kingdom
4. Health Economics and Decision Science, School of Health and Related Research, University of Sheffield, S1 4DA, UK

Corresponding authors:

- Professor James Catto, Academic Urology Unit, G Floor, The Medical School, University of Sheffield, Sheffield, UK. Tel: +44 (0)114 226 1229; Email: j.catto@sheffield.ac.uk and
- Professor Peter Sasieni, King's College London Faculty of Life Sciences & Medicine, School of Cancer & Pharmaceutical Sciences, Cancer Prevention Group, Guy's Campus, UK. Email: peter.sasieni@kcl.ac.uk

Running title: **YORKSURe study for early detection of bladder cancer**

Abstract word count: 300

Word count: 3,522

Keywords: Bladder cancer; screening; early-detection; haematuria

For peer review only

Abstract

Introduction

Around 25% of patients with bladder cancer (BCa) present with invasive disease. Non-randomised studies of population-based screening have suggested reductions in BCa-specific mortality are possible through earlier detection. The low prevalence of lethal disease in the general population means screening is not cost-effective and there is no consensus on the best strategy. Yorkshire has some of the highest mortality rates from BCa in England. We aim to test whether population screening in a region of high mortality risk will lead to a downward stage-migration of aggressive BCa, improved survival and is cost-effective.

Methods and analysis

YORKSURe is a tiered, randomised, multi-cohort study to test the feasibility of a large BCa screening randomised controlled trial. In 3 parallel cohorts, participants will self-test urine (at home) up to 6 times. Results are submitted via a mobile app or freephone. Those with a positive result will be invited for further investigation at community-based early detection clinics or within usual NHS pathways. In Cohort 1 we will post self-testing kits to research engaged participants (n=2,000) embedded within the Yorkshire Lung Screening Trial. In Cohort 2 we will post self-testing kits to 3,000 invitees. Cohort 2 participants will be randomised between haematuria and glycosuria testing using a reveal/conceal design. In Cohort 3 we will post self-testing kits to 500 patients within the NHS pathway for investigation of haematuria. Our primary outcomes are rates of recruitment and randomisation, rates of positive test and acceptability of the design. The study is currently recruiting and scheduled to finish June 2023.

Ethics and dissemination

The study has received the following approvals: London Riverside Research Ethics Committee (22/LO/0018) and Health Research Authority Confidentiality Advisory Group (20/CAG/0009). Results will be made available to funders, NHS Trusts, Clinical Commissioning Groups, Strategic Clinical Networks, and other researchers via publicly accessible scientific journals.

For peer review only

Strengths and limitations

- The multi-cohort approach allows for contemporaneous assessment of each facet of the study whilst permitting the participant pathway to be dynamically optimised.
- The conceal/reveal design offers high compliance and low contamination, but may be concerning to participants.
- Organisational logistics to implement such a study design is exhaustive and relies on collaboration with several industry vendors.
- The acceptability and usability of the study platforms is uncertain.
- The sensitivity of a diagnostic pathway without initial flexible cystoscopy is unknown.

For peer review only

Introduction

Bladder cancer (BCa) is a common malignancy and one of the most expensive to manage [1, 2]. BCa's are best stratified into non-muscle-invasive (NMI) and muscle invasive (MI) cancers. In the UK, around 25% of patients present with MI BCa which requires radical treatment in an attempt at cure [3, 4]. BCa rates increase with age, smoking and occupational carcinogen exposure [5-7]. Consequently, BCa incidence and mortality demographics often match local risk factor patterns of exposure. Yorkshire has some of the highest incidence and mortality rates of BCa in England, perhaps reflecting lifestyle choices and local industries [2, 8,9, 10].

In contrast to many human malignancies, disease specific survival for BCa is not improving [2, 11], suggesting a need to change the approach to manage this cancer. Many BCa's present late to healthcare professionals as the symptoms mirror those of common benign conditions, such as bacterial cystitis, detrusor overactivity or prostatic enlargement [12]. Within the NHS, more BCa's are diagnosed following routine GP referral (41.3% (40.7-42.0%)) for urinary symptoms than via 2 week-wait (2ww) pathways (27.5% (CI: 26.9-28.1%)), and the rate of emergency presentation increases with advancing tumour stage, from 6.4% for NMI to 18.8% for MI BCa's [2].

Current knowledge: Bladder Cancer Early Detection

Most BCa's are diagnosed in persons with visible haematuria (VH) or non-visible haematuria (NVH) [13]. As such, there is potential to use haematuria to detect asymptomatic disease earlier than would occur otherwise [14]. Indeed, patient surveys reveal many individuals complain of irritative bladder symptoms (dysuria, frequency) prior to developing VH [15]. However, there have been few attempts at screening for BCa using haematuria. Two non-randomised single arm studies found haematuria testing in asymptomatic persons leads to earlier stage of cancers at detection and may improve survival [16, 17]. Britton et al. screened 2,356 British men and found 17 BCa's, of which all were NMI [18]. At 7 years, 33% of the high grade NMI cohort had died from BCa, reflecting potential under-management of this aggressive cancer [19]. Messing et al. screened 1,575 American men and found a lower stage at diagnosis, with reduced BCa mortality rate (0%) in screened men compared with rates in men from a matching state-wide registry (16.4% mortality rate) [17]. The authors concluded that screening did reduce mortality, but it needs to be applied in high-risk populations and that haematuria

For peer review only - http://bmjopen.bmj.com/site/about/guidelines.xhtml

is intermittent in BCa patients [20]. In populations exposed to aristolochic acid, Zlotta et al. found survival (0% mortality) occurred only with screening (100% mortality in non-screened patients) [21], supporting the potential for BCa screening to be cost-effective in high-risk populations [22].

Research Need

YORKSURe is a feasibility trial aimed at the early diagnosis of BCa through the detection of haematuria in asymptomatic individuals at high risk of mortality from the cancer. Following results of the feasibility trial, the aim is to conduct a large-scale trial, with sufficient power to test any differences in bladder cancer mortality. However, to undertake this successfully, we need to understand if our proposed approach is robust, appropriate, necessary, and acceptable to participants.

Study aims and objectives

Three cohorts will be studied in this feasibility study. Each will address a separate aim, all of which are needed to develop a phase 3 prospective trial:

Aim 1: Cohort 1 will test compliance, prevalence of haematuria and BCa, and the economics of the trial within a screening-engaged population. It might suggest that BCa screening works best when embedded within lung screening services. It will also test women and populations younger than typical for the highest risk BCa age group.

Aim 2: Cohort 2 will test compliance, prevalence of haematuria and BCa, prevalence of glycosuria and undiagnosed diabetes within a high-risk community-based population.

Aim 3: Cohort 3 will provide a positive predictive value of the study tests (urine self-testing, urine cytology and ultrasound) as assessed in a population already referred in the NHS 2 week wait haematuria pathway.

For peer review only - http://bmjopen.bmj.com/site/about/guidelines.xhtml

Methods and analysis

Study design and setting

Eligibility for Cohort 1: Men and Women aged 55 – 80 years who are registered with a GP within the Leeds Clinical Commissioning Group area, who are part of the Yorkshire Lung Screening Trial (YLST) [23] and have given consent to be contacted by other research teams (figure 1).

Eligibility for Cohort 2: Men aged 65-79 identified through a database search at 8-10 selected GP practices considered to be in high-risk BCa mortality areas in South Yorkshire (figure 2).

Eligibility for Cohort 3: Men and woman will be identified via local urology departments when a referral is made for a 2 week-wait urology appointment for investigation of haematuria *(figure 3)*.

Exclusion criteria:

Cohort 1:

- Unable to or did not consent to contact from other research teams.
- Insufficient capacity to give consent to take part in the research as determined by the investigator taking consent.

Cohort 2:

- Participant has opted out of their NHS data being used for research via the National Data Opt-Out.
- Prior diagnosis of bladder or kidney cancer.
- Diagnosis with any other cancer within the past 5 years (except non-melanoma skin cancer).
- Insufficient capacity to give consent to take part in the research, defined as the presence of the following conditions coded in the GP records; dementia, Alzheimer's disease, or Parkinson's disease; or as determined by the investigator taking consent.

Cohort 3:

- Unable to provide written informed consent.
- Insufficient capacity to give consent to take part in the research, defined as the presence of the following conditions coded in the GP records; dementia, Alzheimer's disease, or Parkinson's disease; or as determined by the investigator taking consent.

For peer review only

- Evidence in patient medical record of historic dissent to use of data in research or participant has opted out of data use via the National Data Opt-Out.

Screening tests and self-testing

Participants in all three cohorts will be provided with home urine self-testing kits produced and supplied by Testcard™. The kit contains 6 x Roche Combur 5 HC test strips for the graduated determination of glucose, leukocytes, nitrites, protein and erythrocytes. Blood and glucose will be tested for primary study purposes. The other three parameters (leukocytes, nitrite, protein) will be analysed as part of an exploratory objective.

Participants will record 6 continuous days of test strip results via a bespoke study App or study freephone service. Prior to commencing self-testing, participants will complete a bladder symptom questionnaire (a modified and unvalidated version of the Urinary Tract Infection Symptom Assessment questionnaire (UTISA) [24]. The questionnaire will collect demographic data and information on bladder symptoms within the past 4 weeks including presence of VH. The questionnaire will be pseudonymised and all data pertaining to the questionnaire will be kept separate from urine self-testing results until the end of the trial. The app will capture a digital photograph of the test strip, which will be interpreted by a research nurse against the Roche Combur 5 HC colour chart. Test strip results will not be available to participants at the point of self-testing.

It is anticipated and expected that some participants in the study population will either not have a smartphone, or will not be confident in using a mobile App. A study freephone service will therefore be available as an alternative option for recording self-test results. The freephone service will be an automated system whereby participants use their keypad to enter their responses to the questionnaire and test-strip results. The freephone service requires confirmation of the test strip colour result by the participant against the Roche Combur 5 HC colour chart, whereas App users will simply take a photograph of their completed test strip which will be submitted for interpretation by a research nurse.

For peer review only

For peer review only - http://bmjopen.bmj.com/site/about/guidelines.xhtml

Investigation of haematuria

Participants in cohort 1 and those randomised to the haematuria arm of cohort 2 will be investigated for the presence of haematuria (figures 1 and 2). If a single positive result is identified upon research nurse review of the erythrocyte test parameter, then the participant will be invited to attend an Early Detection Clinic (run in a nearby GP surgery and staffed by study-employed or CRN employed nursing staff) within 4 – 6 weeks of assessment of their results. At this clinic, participants will undergo a urinary tract ultrasound scan (USS) and provide a urine sample for cytology. Those with an abnormal USS (bladder or renal mass suggestive of cancer detected) or abnormal cytology, will be referred by their GP or the research nurse team (following standard NHS 2-week-wait criteria) to their local urology haematuria service for evaluation (flexible cystoscopy and additional assessments). We expect that around 20% of participants with self-testing haematuria will require referral. The test results for these participants will be obtained directly through the relevant urology departments and categorised into: BCa diagnosis, kidney cancer diagnosis, upper tract urothelial cancer diagnosis, urinary stones, infection or inflammation, stricture, clinically benign prostatic enlargement, post-TURP (transurethral resection of the prostate), diagnosis unclear/further tests planned, likely prostate cancer, no abnormal finding, or other finding. Participants with normal USS and normal cytology, from the early detection clinic, will receive a letter explaining this and discharging them from the study. They will receive advice about the need to see their GP if they have urinary symptoms or VH. Their GP will receive a letter with advice about management of non-visible haematuria. Participants without haematuria will receive a letter confirming their result and informed that their GP will also be notified of their results. They will be asked to seek a GP appointment if they develop urinary symptoms in the future. Participants in cohort 3 will undergo standard NHS assessment of haematuria as part of their referral process (regardless of their test strip results). This includes cystoscopy and upper tract imaging (typically CT urogram and USS).

Investigation of glycosuria

Participants randomised to the glycosuria arm of cohort 2 will be investigated for the presence of glycosuria. Glycosuria was chosen as it is a validated urine test that can detect a meaningful disease (diabetes mellitus), whose outcomes are better detected at an early stage. If a single positive result is identified upon research nurse review of the glucose test

For peer review only

parameter, then the participant will be invited to attend an Early Detection Clinic within 4 – 6 weeks of assessment of their results. At the clinic, participants will undergo HbA1c blood testing. Those with elevated HbA1c (as defined by local reference ranges) will be referred to their GP for diabetic management. Those with a normal HbA1c (as defined by local reference ranges) will receive a letter explaining that their test was normal and will be advised to see their GP if they think they have diabetic symptoms. Their GP will also be informed of the test result. Participants in the glucosuria testing arm without glycosuria will receive a letter confirming their normal result and informed that their GP will also be notified of their results. Participants will be advised to seek a GP appointment if they develop diabetic symptoms.

Consent

Upon receipt of the postal urine self-testing kit, participants will be able to choose an action to take:

1. Consent to enter trial: Those wishing to take part in the trial will be asked to consent to participate by agreeing to consent statements in the mobile App or freephone system, before proceeding to complete the study questionnaire, and then self-testing with the test strips. Invitees will be prompted prior to providing results to have read the Participant Information Sheet.
2. Non-response: non-responders (i.e., do not consent or provide test results via the mobile App or freephone)

Following a positive urine self-test result for haematuria (Cohort 1) and haematuria/glucosuria (Cohort 2) participants will receive a telephone call from a study research nurse to explain their results and schedule an Early Detection Clinic appointment. This will be followed by the mailout of a second PIS detailing what will happen at the Early Detection Clinic appointment. At the clinic, written informed consent will be obtained prior to any trial specific procedures. The CI will ensure that those with delegated responsibility to obtain informed consent are duly authorised, trained and competent according to the ethically approved protocol, principles of GCP and Declaration of Helsinki. The right of a participant to refuse participation without giving reasons will be respected.

For peer review only

For peer review only - http://bmjopen.bmj.com/site/about/guidelines.xhtml

Cohort 3 participants will receive a self-testing kit including a study invitation letter and PIS detailing what will happen at the 2ww appointment (figure 3). The invitation letter will be from the routine care team at the participating urology department. On presentation to the 2ww appointment, written informed consent will be obtained prior to any trial specific procedures. Translation via hospital interpreters is acceptable if required. The Principal Investigator (PI) will ensure that those with delegated responsibility to obtain informed consent are duly authorised, trained and competent according to the ethically approved protocol, principles of GCP and Declaration of Helsinki. The right of a participant to refuse participation without giving reasons will be respected. For Cohort 3, participation is mostly altruistic as the participants will already be in the NHS pathway and will have the gold-standard tests for detection of BCa.

Sample size(s)

A formal power calculation is not appropriate for this trial due to its feasibility related outcomes. The current planned sample size is to invite 2,000 participants in Cohort 1, (estimated 800 enrolled), invite 3,000 male participants in Cohort 2, (estimated to enrol and randomise 1,200) and invite 1,000 participants in Cohort 3 (estimated enrolment of 500).

Randomisation

Cohort 2 will be randomised between two arms where either the haematuria or glycosuria result will be revealed during the trial. Participants will be randomised via permuted blocks to ensure approximately equal numbers in the two arms.

Data collection

Self-test results and questionnaire data will be captured in the bespoke mobile app and freephone system. The questionnaire will collect information on diabetes (diagnosis and management), smoking status, ethnic group, LGBT identification, highest level of education, history of factory work as well as bladder symptoms (via a modified and unvalidated version of the Urinary Tract Infection Symptom Assessment questionnaire (UTISA) [24]). Cohort 1 and 2 data collected at Early Detection Clinics will be entered into a REDCap database and will include height and weight, current occupation, previous longest occupation, concomitant medication, ultrasound results, urine cytology results, and HbA1c results. Data for Cohort 1

For peer review only - http://bmjopen.bmj.com/site/about/guidelines.xhtml
For peer review only

and 2 participants that are subsequently referred for a 2WW, and consenting Cohort 3 participants, will be obtained via medical records to include results of cystoscopy, ultrasound/CT, cytology, and cancer staging where applicable. At 14-30 days post cystoscopy, these participants will be contacted to collect information of any treated incidences of UTI following cystoscopy. Passive follow-up data (identification of cancer diagnosis and stage) will be collected through the National Cancer Registration and Analysis Service (NCRAS) at 1, 2, and 3 years after the end of the trial for all participants that have self-tested (excluding withdrawals). Summary data will also be collected through NCRAS, requested by geographic area, for a population comparable to Cohort 2.

Statistical Analysis

We will present estimates of percentages, with 95% Wilson confidence intervals, on a per cohort basis for the three trial endpoints that will allow assessment of feasibility and inform the subsequent more definitive trial. We will estimate acceptability (proportion of invitees consenting and returning a test strip), compliance (proportion of participants returning at least 3 or all 6 test strips), haematuria and glucosuria, proportion of positive outcomes for those referred to the Early Detection Clinic (positive cytology and/or USS for haematuria and HbA1c for glycosuria). We will assess the levels of BCa in the cohort identified by Early Detection clinic and at 3-years using National Disease Registration Service (NDRS, formerly NCRAS) and the accuracy of the haematuria test results including positive and negative predictive values (NPV and PPV) for cohorts 1 and 2 only. The urinary symptoms questionnaires will be summarised by cohort, by the proportions with each urinary symptom, and by overall urinary symptoms using mean, SD, median, minimum, maximum and quartiles. Missing values will be imputed as no symptom.

Economic evaluation

An early health economic model of haematuria screening for bladder cancer in high-risk areas will be developed, including epidemiology, natural history and how this is affected by screening. The model design will be informed through analysis of other screening and diagnostic models in BCa. The model will be parameterised with the generated feasibility outcome data, supplemented by evidence from published literature. Bayesian calibration

For peer review only - http://bmjopen.bmj.com/site/about/guidelines.xhtml

methods will be used for parameterisation of the disease natural history and screening model. The model will input to the design of and be refined using the subsequent trial outcomes.

Patient and public involvement (PPI)

A group of 9 PPI representatives were approached for initial review of the trial design, objectives, lay summary, and survey questions, and their responses have been incorporated in the trial design. PPI members have also reviewed the participant facing documentation and provided feedback. One-to-one interviews and discussions with PPI members were held to obtain specific feedback on aspects of how the study is presented to potential participants. A PPI lead is included in the Trial Steering Committee.

Trial organisation and administration

The trial is sponsored by the University of Sheffield and coordinated by the Cancer Research UK & King's College London Cancer Prevention Trials Unit (CPTU). The central research nursing team and central cytology laboratory is based at Sheffield Teaching Hospital NHS Foundation Trust.

Two trial oversight groups provide guidance and management for the three cohorts. The Trial Management Group meet at least monthly and will comprise the Chief Investigator, representatives from University of Sheffield, and CPTU who are responsible for day-to-day management of the trial and carrying out the work detailed in the trial protocol. The purpose of the group is to ensure the trial is on track to its project plan and budget, manage trial risks, issues, and mitigations and to escalate and report any major risks and issues to the Trial Steering Committee (TSC) as necessary.

The TSC will concentrate on progress of the trial, adherence to the protocol, participant welfare and to assess the impact and relevance of any new information of relevance to the research question. The TSC will make recommendations, through its Chair, to the TMG, Chief Investigator, trial sponsor, trial funders, CPTU and any other relevant party on all appropriate aspects of the trial. Most members of the TSC, including the Chair, will be independent of the trial, and will include lay/patient representation. Non-independent members will also be part

For peer review only

of the TSC as observers. The final decision regarding whether the trial may continue is the responsibility of the TSC.

Ethics and Dissemination

A risk assessment and review of ethical considerations has been performed as part of the trial, including development of mitigation strategies to ensure the safe and successful delivery of the trial. Key risks and mitigations from this trial have been outlined below:

- Cancer screening is in part controversial due to the risks of finding and over treating indolent cancers. However, for BCa there is minimal morbidity in diagnosis (i.e., from the TURBT) and risk stratification is embedded in the treatment pathways. Many of these cancers are managed without radical treatment, and therefore there are few long-term side effects. Furthermore, autopsy studies [9] reveal few undiagnosed BCa's, suggesting that most tumours present clinically. Modelling of the disease natural history will aim to estimate overdiagnosis and will provide input to subsequent trial design to provide definitive evidence on these issues.
- Another risk is that participants may attempt to seek the same test outside of the trial or try to identify what the colour change on their urinary test strip represents. We have worked with PPI representatives to tailor trial communications. It is key that participants understand that participation in the trial should be seen as altruistic and there is currently no evidence to suggest that screening of this may be beneficial and indeed may cause harm. Liaison with local GPs throughout the pilot period will be key in determining if they observe any increase in men or women requesting urinalysis assessments.
- Variable practices in secondary care for the treatment of BCa (i.e., post study) is another risk. Treating clinicians may use a wide variety of different regimens) to manage screen detected cancers. As part of the planned future trial, we will apply for funding for an embedded cohort study of comparative effectiveness and health-related quality of life (HRQOL) for these screen-detected cancers. Patients/clinicians will be able to choose from a selection of standard National Institute of Care Excellence (NICE) approved pathways. HRQOL and cost will be collected within these pathways.

To ensure widespread dissemination, we plan to circulate the trial results to multiple research outputs including but not limited to conference proceedings and peer-reviewed journal

For peer review only

For peer review only - http://bmjopen.bmj.com/site/about/guidelines.xhtml

articles relating to the trial design, results of the pilot trial, final uptake of participants and a final main analysis of primary and secondary endpoints encompassing economic modelling aspects. All trials will be published in high-impact journals, accompanied by a press release. This will be covered in more detail in the trial publication policy. Dissemination of trial results will be designed and delivered after extensive collaboration with PPI groups.

Trial Status

The trial opened to recruitment on 3rd October 2022 and is in the active recruitment phase.

For peer review only

For peer review only - http://bmjopen.bmj.com/site/about/guidelines.xhtml

Acknowledgements:

The trial is led by Professor James Catto, sponsored by the University of Sheffield, and coordinated by the Cancer Research UK & King's College London Cancer Prevention Trials Unit (CPTU). The trial is supported by TestCard Ltd, InHealth, Sealed Envelope and iPlato Healthcare. The authors would like to thank Joanna Gambell and Harriet Strachan of the Kings College London Cancer Prevention Group for their expertise and assistance with the management, coordination and administration of the YORKSURe study. The authors would also like to thank Allen Knight of Action Bladder Cancer UK and Lydia Makaroff of World Bladder Cancer Patient Coalition/Fight Bladder Cancer for their review and guidance during the development of the study design. Study data were collected and managed using REDCap [25] Research Electronic Data Capture, an electronic, web-based data capture tool hosted at the Cancer Prevention Group, King's College London.

Author contributions:

JWFC – concept, protocol development and manuscript (all)

BN – statistics and manuscript (statistics/trial design)

MG – protocol development, trial management and manuscipt (all)

AC– protocol development, trial management and construction of manuscipt (all)

MS – concept and protocol development

OM – health economic modelling

JCh – concept and health economic modelling

PS – concept, trial design, statistics, and manuscript (all)

MGC – concept, protocol development and manuscipt (all)

Ethics Approvals:

The study has received the following approvals: London – Riverside Research Ethics Committee (22/LO/0018), Health Research Authority Confidentiality Advisory Group (20/CAG/0009).

Participant Consent for Publication:

Not required. No identifiable personal data will be used in publications.

For peer review only

Data Availability:

Upon reasonable requests to the study team, deidentified participant data will be available after publication of the study outcomes. Use and projects need approval by the Trial Steering Committee.

Registration:

The study is registered on the ISRCTN registry: ISRCTN34273159

Competing interests:

JWFC has received reimbursement for consultancy from Astra Zeneca, BMS, Ipsen, Janssen and Roche, speaker fees from BMS, Ipsen, MSD, Nucleix and Roche, honoraria for membership of advisory boards from Astra Zeneca, Ferring, Roche and Janssen, and research funding from Roche. PS is a paid member of the Scientific Advisory Board of GRAIL and the medical advisory board of NSV. The remaining authors declare no potential conflicts of interest.

Funding:

This work was funded by Yorkshire Cancer Research (Grant number RA/2019/R1/004: S431 Early detection of bladder cancer in Yorkshire: Feasibility assessments for implementing a targeted study in populations with high disease specific mortality risk). The funder had no role in the design, analysis or collection of the data; in writing the manuscript; or in the decision to submit the manuscript for publication. JWFC is funded by an NIHR Research Professorship. MGC is funded by the NIHR (senior lecturer) and is in receipt of a RCSEng Hunterian Professorship (2023). BN and MS are funded by a Cancer Research UK grant led by PS (C8162/A25356). The NIHR have funded the service support costs and Part B research costs associated with the study. TestCard Ltd have provided an in-kind donation for the develop of the YORKSURe Mobile App.

For peer review only

References

1. Cumberbatch MGK, Jubber I, Black PC, et al. Epidemiology of Bladder Cancer: A Systematic Review and Contemporary Update of Risk Factors in 2018. *Eur Urol* 2018;74(6):784-95. doi: 10.1016/j.eururo.2018.09.001 [published Online First: 2018/10/01]

2. Catto JWF, Mandrik O, Quayle LA, et al. Diagnosis, treatment and survival from bladder, upper urinary tract and urethral cancers: Real world findings from NHS England between 2013 and 2019. *BJU Int* 2023 doi: 10.1111/bju.15970 [published Online First: 2023/01/22]

3. Catto JWF, Khetrapal P, Ricciardi F, et al. Effect of Robot-Assisted Radical Cystectomy With Intracorporeal Urinary Diversion vs Open Radical Cystectomy on 90-Day Morbidity and Mortality Among Patients With Bladder Cancer: A Randomized Clinical Trial. *JAMA* 2022;327(21):2092-103. doi: 10.1001/jama.2022.7393 [published Online First: 2022/05/16]

4. Catto JWF, Gordon K, Collinson M, et al. Radical Cystectomy Against Intravesical BCG for High-Risk High-Grade Nonmuscle Invasive Bladder Cancer: Results From the Randomized Controlled BRAVO-Feasibility Study. *J Clin Oncol* 2021;39(3):202-14. doi: 10.1200/JCO.20.01665 [published Online First: 2020/12/18]

5. Jubber I, Ong S, Bukavina L, et al. Epidemiology of Bladder Cancer in 2023: A Systematic Review of Risk Factors. *Eur Urol* 2023;In Press

6. Cumberbatch MG, Cox A, Teare D, et al. Contemporary Occupational Carcinogen Exposure and Bladder Cancer: A Systematic Review and Meta-analysis. *JAMA Oncol* 2015;1(9):1282-90. doi: 10.1001/jamaoncol.2015.3209 [published Online First: 2015/10/09]

7. Cumberbatch MG, Rota M, Catto JW, et al. The Role of Tobacco Smoke in Bladder and Kidney Carcinogenesis: A Comparison of Exposures and Meta-analysis of Incidence and Mortality Risks. *Eur Urol* 2016;70(3):458-66. doi: 10.1016/j.eururo.2015.06.042 [published Online First: 2015/07/08]

8. NHS Digital. Cancer incidence and mortality 2021 [National Disease Registration Service: Comparisons by NHS integrated care boards]. Available from: https://www.cancerdata.nhs.uk/incidence_and_mortality accessed 31st January 2023.

9. Catto JWF, Rogers Z, Downing A, et al. Lifestyle factors in patients with bladder cancer: A contemporary picture of tobacco smoking, electronic cigarette use, body mass index and levels of physical activity. . *Eur Urol Focus* 2023;In Press

10. Reed O, Jubber I, Griffin J, et al. Occupational bladder cancer: A cross section survey of previous employments, tasks and exposures matched to cancer phenotypes. *PLoS One* 2020;15(10):e0239338. doi: 10.1371/journal.pone.0239338 [published Online First: 2020/10/22]

For peer review only

11. Eylert MF, Hounsome, L. S.; Persad, R.; Bahl, A.; Jefferies, E.; Verne, J.; Mostafid, H. Falling bladder cancer incidence from 1990 to 2009 is not producing universal mortality improvements. *Journal of Clinical Urology* 2014;7(2):90-98.

12. Lyratzopoulos G, Saunders CL, Abel GA, et al. The relative length of the patient and the primary care interval in patients with 28 common and rarer cancers. *Br J Cancer* 2015;112 Suppl 1:S35-40. doi: 10.1038/bjc.2015.40 [published Online First: 2015/03/04]

13. Jubber I, Shariat SF, Conroy S, et al. Non-visible haematuria for the Detection of Bladder, Upper Tract, and Kidney Cancer: An Updated Systematic Review and Meta-analysis. *Eur Urol* 2020;77(5):583-98. doi: 10.1016/j.eururo.2019.10.010 [published Online First: 2019/12/04]

14. Larre S, Catto JW, Cookson MS, et al. Screening for bladder cancer: rationale, limitations, whom to target, and perspectives. *Eur Urol* 2013;63(6):1049-58. doi: 10.1016/j.eururo.2012.12.062 [published Online First: 2013/01/15]

15. Elliott J, Rotterud R, Maddox-Smith A, et al. The bladder cancer patient survey: Global perspectives on awareness and treatment of bladder cancer. *Journal of Cancer Policy* 2019;22:100207.

16. Britton JP, Dowell AC, Whelan P. Dipstick haematuria and bladder cancer in men over 60: results of a community study. *BMJ* 1989;299(6706):1010-2. doi: 10.1136/bmj.299.6706.1010 [published Online First: 1989/10/21]

17. Messing EM, Young TB, Hunt VB, et al. Comparison of bladder cancer outcome in men undergoing hematuria home screening versus those with standard clinical presentations. *Urology* 1995;45(3):387-96; discussion 96-7. doi: 10.1016/s0090-4295(99)80006-5 [published Online First: 1995/03/01]

18. Mayfield MP, Whelan P. Bladder tumours detected on screening: results at 7 years. *Br J Urol* 1998;82(6):825-8. doi: 10.1046/j.1464-410x.1998.00879.x [published Online First: 1999/01/12]

19. Thomas F, Rosario DJ, Rubin N, et al. The long-term outcome of treated high-risk nonmuscle-invasive bladder cancer: time to change treatment paradigm? *Cancer* 2012;118(22):5525-34. doi: 10.1002/cncr.27587 [published Online First: 2012/05/01]

20. Messing EM, Young TB, Hunt VB, et al. Hematuria home screening: repeat testing results. *J Urol* 1995;154(1):57-61. doi: 10.1016/s0022-5347(01)67224-0 [published Online First: 1995/07/01]

21. Zlotta AR, Roumeguere T, Kuk C, et al. Select screening in a specific high-risk population of patients suggests a stage migration toward detection of non-muscle-invasive bladder cancer. *Eur Urol* 2011;59(6):1026-31. doi: 10.1016/j.eururo.2011.03.027 [published Online First: 2011/04/05]

For peer review only

22. Mandrik O, Hahn AI, Catto JWF, et al. Critical Appraisal of Decision Models Used for the Economic Evaluation of Bladder Cancer Screening and Diagnosis: A Systematic Review. *PharmacoEconomics* 2023;In press doi: 10.1007/s40273-023-01256-9

23. Crosbie PA, Gabe R, Simmonds I, et al. Yorkshire Lung Screening Trial (YLST): protocol for a randomised controlled trial to evaluate invitation to community-based low-dose CT screening for lung cancer versus usual care in a targeted population at risk. *BMJ Open* 2020;10(9):e037075. doi: 10.1136/bmjopen-2020-037075 [published Online First: 2020/09/12]

24. Clayson D, Wild D, Doll H, et al. Validation of a patient-administered questionnaire to measure the severity and bothersomeness of lower urinary tract symptoms in uncomplicated urinary tract infection (UTI): the UTI Symptom Assessment questionnaire. *BJU Int* 2005;96(3):350-9. doi: 10.1111/j.1464-410X.2005.05630.x [published Online First: 2005/07/27]

25. Harris PA, Taylor R, Thielke R, et al. Research electronic data capture (REDCap)--a metadata-driven methodology and workflow process for providing translational research informatics support. *J Biomed Inform* 2009;42(2):377-81. doi: 10.1016/j.jbi.2008.08.010 [published Online First: 2008/10/22]

For peer review only

For peer review only - http://bmjopen.bmj.com/site/about/guidelines.xhtml

Figures

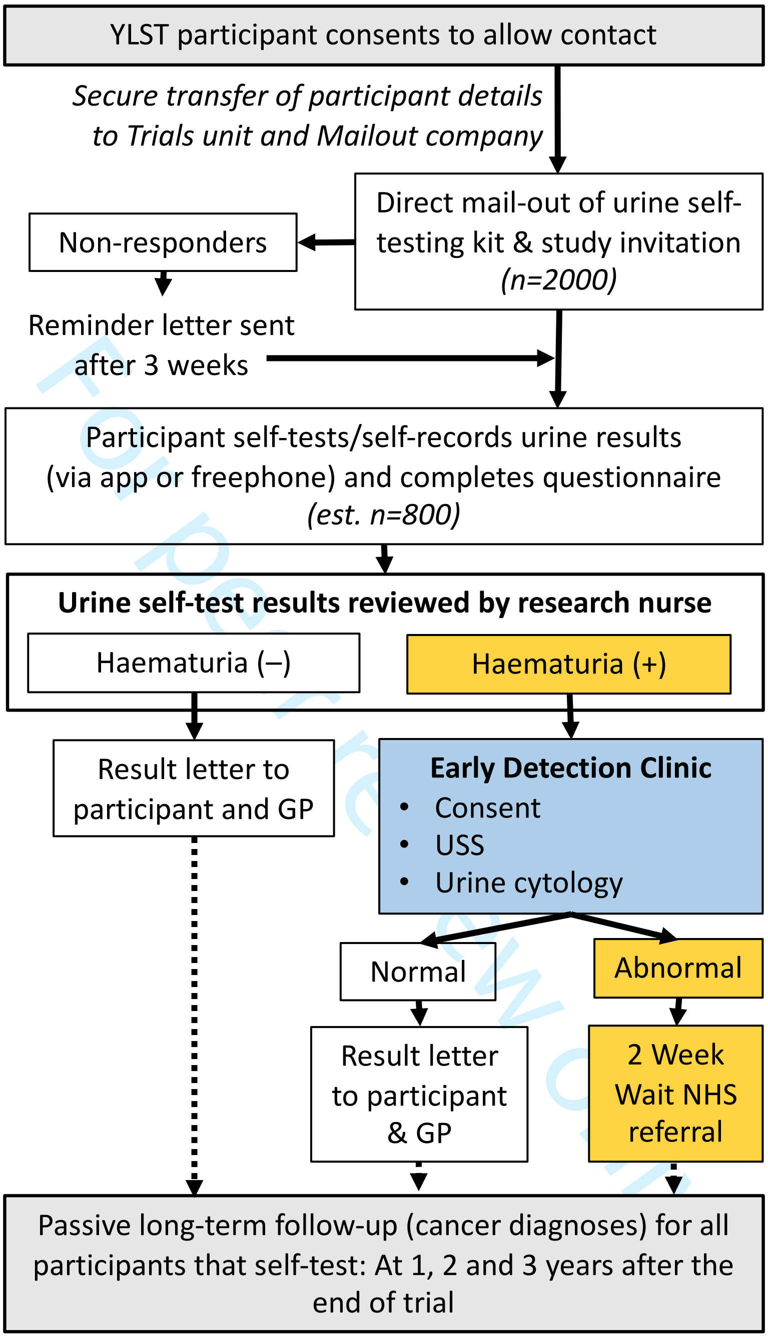

Figure 1. Recruitment and flow within Cohort 1 within the YORKSURe study: Male and Female participants will be solely recruited from the Yorkshire Lung Screening Trial (YLST).

For peer review only - http://bmjopen.bmj.com/site/about/guidelines.xhtml

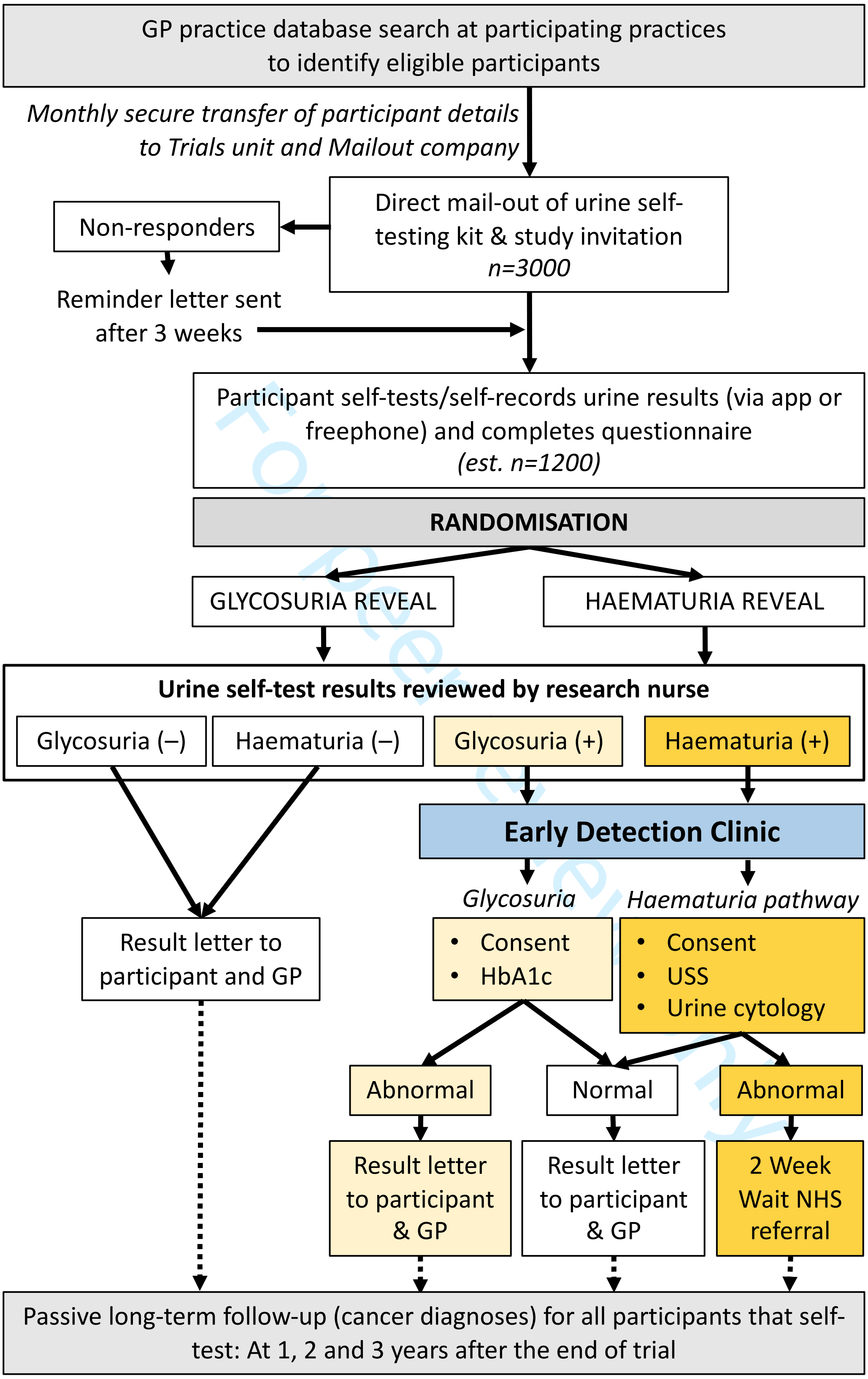

Figure 2. Recruitment and flow within Cohort 2 of the YORKSURe study: Male participants will be invited from populations at risk of high-mortality from bladder cancer.

For peer review only - http://bmjopen.bmj.com/site/about/guidelines.xhtml

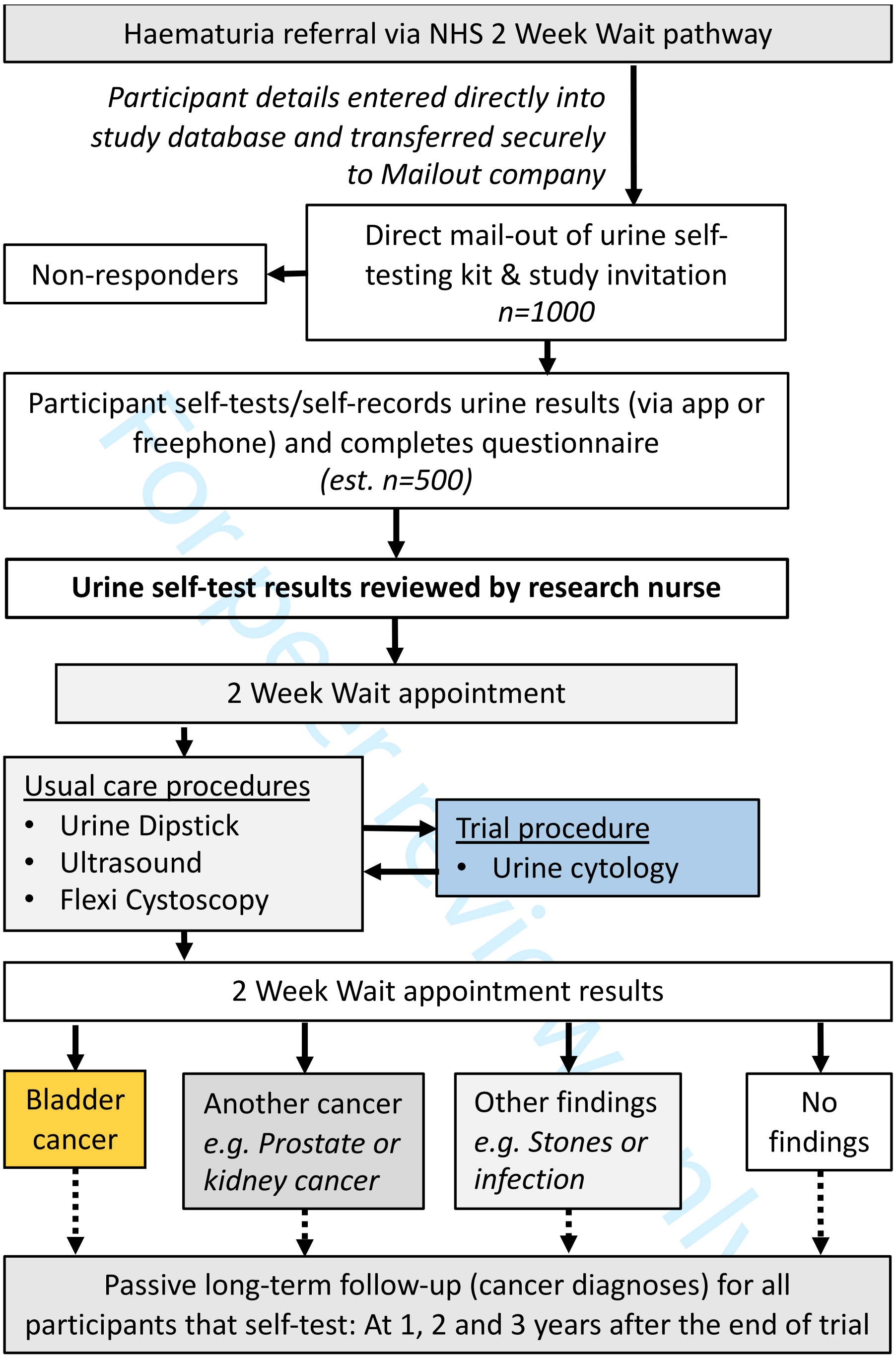

Figure 3. Participant flow within Cohort 3 of the YORKSURe study: Male and female patients undergoing investigation within the NHS for haematuria (according to the 2 week wait criteria within the NICE guidelines) will be invited to participate.

For peer review only - http://bmjopen.bmj.com/site/about/guidelines.xhtml

BMJ Open

Protocol for the YORKSURe prospective multi-stage study testing the feasibility for early detection of bladder cancer in populations with high disease specific mortality risk

Journal:	*BMJ Open*
Manuscript ID	bmjopen-2023-076612.R1
Article Type:	Protocol
Date Submitted by the Author:	11-Jul-2023
Complete List of Authors:	Catto, James; University of Sheffield Medical School, Oncology and Metabolism<br>North, Bernard; King's College London,<br>Goff, Megan; King's College London<br>Carter, Abigail; King's College London<br>Sleeth, Michelle; King's College London<br>Mandrik, Olena; The University of Sheffield,<br>Chilcott, Jim; University of Sheffield, Health Economics and Decision Science<br>Sasieni, Peter; King's College London<br>Cumberbatch, Marcus; The University of Sheffield, Academic Urology
**Primary Subject Heading**:	Urology
Secondary Subject Heading:	Oncology, Surgery, Smoking and tobacco, Public health
Keywords:	Bladder disorders < UROLOGY, Mass Screening, Urological tumours < ONCOLOGY

SCHOLARONE™
Manuscripts

Protocol for the YORKSURe prospective multi-stage study testing the feasibility for early detection of bladder cancer in populations with high disease specific mortality risk

James W.F. Catto [1,2], Bernard North [3], Megan Goff [3], Abigail Carter [3], Michelle Sleeth [3], Olena Mandrik [4], Jim Chilcott [4], Peter Sasieni [3*] and Marcus G Cumberbatch [1,2*]

* Shared senior authorship

James Catto [Orcid ID = orcid.org/0000-0003-2787-8828]

Peter Sasieni [Orcid ID = orcid.org/0000-0003-1509-8744]

Affiliations:

1. Academic Urology Unit, University of Sheffield, Sheffield, UK; Department of Urology, Sheffield Teaching Hospitals NHS Trust, Sheffield, UK.
2. Department of Urology, Sheffield Teaching Hospitals NHS Foundation Trust, Sheffield
3. Cancer Prevention Group, School of Cancer and Pharmaceutical Sciences, Faculty of Life Sciences and Medicine, King's College London, London, United Kingdom
4. Health Economics and Decision Science, School of Health and Related Research, University of Sheffield, S1 4DA, UK

Corresponding authors:

- Professor James Catto, Academic Urology Unit, G Floor, The Medical School, University of Sheffield, Sheffield, UK. Tel: +44 (0)114 226 1229; Email: j.catto@sheffield.ac.uk

and

- Professor Peter Sasieni, King's College London Faculty of Life Sciences & Medicine, School of Cancer & Pharmaceutical Sciences, Cancer Prevention Group, Guy’s Campus, UK. Email: peter.sasieni@kcl.ac.uk

Running title: **YORKSURe study for early detection of bladder cancer**

Abstract word count: 298

Word count: 4,206

Keywords: Bladder cancer; screening; early-detection; haematuria

For peer review only - http://bmjopen.bmj.com/site/about/guidelines.xhtml

Abstract

Introduction

Around 25% of patients with bladder cancer (BCa) present with invasive disease. Non-randomised studies of population-based screening have suggested reductions in BCa-specific mortality are possible through earlier detection. The low prevalence of lethal disease in the general population means screening is not cost-effective and there is no consensus on the best strategy. Yorkshire has some of the highest mortality rates from BCa in England. We aim to test whether population screening in a region of high mortality risk will lead to a downward stage-migration of aggressive BCa, improved survival and is cost-effective.

Methods and analysis

YORKSURe is a tiered, randomised, multi-cohort study to test the feasibility of a large BCa screening randomised controlled trial. In 3 parallel cohorts, participants will self-test urine (at home) up to 6 times. Results are submitted via a mobile app or freephone. Those with a positive result will be invited for further investigation at community-based early detection clinics or within usual NHS pathways. In Cohort 1 we will post self-testing kits to research engaged participants (n=2,000) embedded within the Yorkshire Lung Screening Trial. In Cohort 2 we will post self-testing kits to 3,000 invitees. Cohort 2 participants will be randomised between haematuria and glycosuria testing using a reveal/conceal design. In Cohort 3 we will post self-testing kits to 500 patients within the NHS pathway for investigation of haematuria. Our primary outcomes are rates of recruitment and randomisation, rates of positive test and acceptability of the design. The study is currently recruiting and scheduled to finish June 2023.

Ethics and dissemination

The study has received the following approvals: London Riverside Research Ethics Committee (22/LO/0018) and Health Research Authority Confidentiality Advisory Group (20/CAG/0009). Results will be made available to providers and researchers via publicly accessible scientific journals.

Registration

The trial is registered as ISRCTN34273159.

For peer review only

Strengths and limitations

- The multi-cohort approach allows for contemporaneous assessment of each facet of the study whilst permitting the participant pathway to be dynamically optimised.
- The conceal/reveal design offers high compliance and low contamination, but may be concerning to participants.
- Organisational logistics to implement such a study design is exhaustive and relies on collaboration with several industry vendors.
- The acceptability and usability of the study platforms is uncertain.
- The sensitivity of a diagnostic pathway without initial flexible cystoscopy is unknown.

For peer review only

For peer review only - http://bmjopen.bmj.com/site/about/guidelines.xhtml

Introduction

Bladder cancer (BCa) is a common malignancy and one of the most expensive to manage [1, 2]. BCa's are best stratified into non-muscle-invasive (NMI) and muscle invasive (MI) cancers. In the UK, around 25% of patients present with MI BCa which requires radical treatment in an attempt at cure [3, 4]. BCa rates increase with age, smoking and occupational carcinogen exposure [5-7]. Consequently, BCa incidence and mortality demographics often match local risk factor patterns of exposure. Yorkshire has some of the highest incidence and mortality rates of BCa in England, perhaps reflecting lifestyle choices and local industries [2, 8],[9, 10].

In contrast to many human malignancies, disease specific survival for BCa is not improving [2, 11], suggesting a need to change the approach to manage this cancer. Many BCa's present late to healthcare professionals as the symptoms mirror those of common benign conditions, such as bacterial cystitis, detrusor overactivity or prostatic enlargement [12]. Within the NHS, more BCa's are diagnosed following routine GP referral (41.3% (40.7-42.0%)) for urinary symptoms than via 2 week-wait (2ww) pathways (27.5% (CI: 26.9-28.1%)), and the rate of emergency presentation increases with advancing tumour stage, from 6.4% for NMI to 18.8% for MI BCa's [2].

Current knowledge: Bladder Cancer Early Detection

Most BCa's are diagnosed in persons with visible haematuria (VH) or non-visible haematuria (NVH) [13]. As such, there is potential to use haematuria to detect asymptomatic disease earlier than would occur otherwise [14]. Indeed, patient surveys reveal many individuals complain of irritative bladder symptoms (dysuria, frequency) prior to developing VH [15, 16]. However, there have been few attempts at screening for BCa using haematuria. Two non-randomised single arm studies found haematuria testing in asymptomatic persons leads to earlier stage of cancers at detection and may improve survival [17, 18]. Britton et al. screened 2,356 British men and found 17 BCa's, of which all were NMI [19]. At 7 years, 33% of the high grade NMI cohort had died from BCa, reflecting potential under-management of this aggressive cancer [20]. Messing et al. screened 1,575 American men and found a lower stage at diagnosis, with reduced BCa mortality rate (0%) in screened men compared with rates in men from a matching state-wide registry (16.4% mortality rate) [18]. The authors concluded that screening did reduce mortality, but it needs to be applied in high-risk populations and

For peer review only - http://bmjopen.bmj.com/site/about/guidelines.xhtml

that haematuria is intermittent in BCa patients [21]. In populations exposed to aristolochic acid, Zlotta et al. found survival (0% mortality) occurred only with screening (100% mortality in non-screened patients) [22], supporting the potential for BCa screening to be cost-effective in high-risk populations [23].

Research Need

YORKSURe is a feasibility trial aimed at the early diagnosis of BCa through the detection of haematuria in asymptomatic individuals at high risk of mortality from the cancer. Following results of the feasibility trial, the aim is to conduct a large-scale trial, with sufficient power to test any differences in bladder cancer mortality. However, to undertake this successfully, we need to understand if our proposed approach is robust, appropriate, necessary, and acceptable to participants. Here we detail the trail using Protocol Vsn. 5.0 date 16/1/2023.

Study aims and objectives

Three cohorts will be studied in this feasibility study. Each will address a separate aim, all of which are needed to develop a phase 3 prospective trial:

Aim 1: Cohort 1 will test compliance, prevalence of haematuria and BCa, and the economics of the trial within a screening-engaged population. It might suggest that BCa screening works best when embedded within lung screening services. It will also test women and populations younger than typical for the highest risk BCa age group.

Aim 2: Cohort 2 will test compliance, prevalence of haematuria and BCa, prevalence of glycosuria and undiagnosed diabetes within a high-risk community-based population.

Aim 3: Cohort 3 will provide a positive predictive value of the study tests (urine self-testing, urine cytology and ultrasound) as assessed in a population already referred in the NHS 2 week wait haematuria pathway.

For peer review only - http://bmjopen.bmj.com/site/about/guidelines.xhtml

Methods and analysis

Study design and setting

Eligibility for Cohort 1: Men and Women aged 55 – 80 years who are registered with a GP within the Leeds Clinical Commissioning Group area, who are part of the Yorkshire Lung Screening Trial (YLST) [24] and have given consent to be contacted by other research teams (figure 1).

Eligibility for Cohort 2: Men aged 65-79 identified through a database search at 8-10 selected GP practices considered to be in high-risk BCa mortality areas in South Yorkshire (figure 2).

Eligibility for Cohort 3: Men and woman will be identified via local urology departments when a referral is made for a 2 week-wait urology appointment for investigation of haematuria *(figure 3)*.

Exclusion criteria:

Cohort 1:

- Unable to or did not consent to contact from other research teams.
- Insufficient capacity to give consent to take part in the research as determined by the investigator taking consent.

Cohort 2:

- Participant has opted out of their NHS data being used for research via the National Data Opt-Out.
- Prior diagnosis of bladder or kidney cancer.
- Diagnosis with any other cancer within the past 5 years (except non-melanoma skin cancer).
- Insufficient capacity to give consent to take part in the research, defined as the presence of the following conditions coded in the GP records; dementia, Alzheimer's disease, or Parkinson's disease; or as determined by the investigator taking consent.

Cohort 3:

- Unable to provide written informed consent.
- Insufficient capacity to give consent to take part in the research, defined as the presence of the following conditions coded in the GP records; dementia, Alzheimer's disease, or Parkinson's disease; or as determined by the investigator taking consent.

For peer review only - http://bmjopen.bmj.com/site/about/guidelines.xhtml

- Evidence in patient medical record of historic dissent to use of data in research or participant has opted out of data use via the National Data Opt-Out.

Screening tests and self-testing

Participants in all three cohorts will be provided with home urine self-testing kits produced and supplied by Testcard™. The kit contains 6 x Roche Combur 5 HC test strips for the graduated determination of glucose, leukocytes, nitrites, protein and erythrocytes. Blood and glucose will be tested for primary study purposes. The other three parameters (leukocytes, nitrite, protein) will be analysed as part of an exploratory objective.

Participants will record 6 continuous days of test strip results via a bespoke study App or study freephone service. Prior to commencing self-testing, participants will complete a bladder symptom questionnaire (a modified and unvalidated version of the Urinary Tract Infection Symptom Assessment questionnaire (UTISA) [25]. The questionnaire will collect demographic data and information on bladder symptoms within the past 4 weeks including presence of VH. The questionnaire will be pseudonymised and all data pertaining to the questionnaire will be kept separate from urine self-testing results until the end of the trial. The app will capture a digital photograph of the test strip, which will be interpreted by a research nurse against the Roche Combur 5 HC colour chart. Test strip results will not be available to participants at the point of self-testing.

It is anticipated and expected that some participants in the study population will either not have a smartphone, or will not be confident in using a mobile App. A study freephone service will therefore be available as an alternative option for recording self-test results. The freephone service will be an automated system whereby participants use their keypad to enter their responses to the questionnaire and test-strip results. The freephone service requires confirmation of the test strip colour result by the participant against the Roche Combur 5 HC colour chart, whereas App users will simply take a photograph of their completed test strip which will be submitted for interpretation by a research nurse.

For peer review only - http://bmjopen.bmj.com/site/about/guidelines.xhtml

Investigation of haematuria

Participants in cohort 1 and those randomised to the haematuria arm of cohort 2 will be investigated for the presence of haematuria (figures 1 and 2). If a single positive result is identified upon research nurse review of the erythrocyte test parameter, then the participant will be invited to attend an Early Detection Clinic (run in a nearby GP surgery and staffed by study-employed or CRN employed nursing staff) within 4 – 6 weeks of assessment of their results. At this clinic, participants will undergo a urinary tract ultrasound scan (USS) and provide a urine sample for cytology. Those with an abnormal USS (bladder or renal mass suggestive of cancer detected) or abnormal cytology, will be referred by their GP or the research nurse team (following standard NHS 2-week-wait criteria) to their local urology haematuria service for evaluation (flexible cystoscopy and additional assessments). We expect that around 20% of participants with self-testing haematuria will require referral. The test results for these participants will be obtained directly through the relevant urology departments and categorised into: BCa diagnosis, kidney cancer diagnosis, upper tract urothelial cancer diagnosis, urinary stones, infection or inflammation, stricture, clinically benign prostatic enlargement, post-TURP (transurethral resection of the prostate), diagnosis unclear/further tests planned, likely prostate cancer, no abnormal finding, or other finding. Participants with normal USS and normal cytology, from the early detection clinic, will receive a letter explaining this and discharging them from the study. They will receive advice about the need to see their GP if they have urinary symptoms or VH. Their GP will receive a letter with advice about management of non-visible haematuria. Participants without haematuria will receive a letter confirming their result and informed that their GP will also be notified of their results. They will be asked to seek a GP appointment if they develop urinary symptoms in the future. Participants in cohort 3 will undergo standard NHS assessment of haematuria as part of their referral process (regardless of their test strip results). This includes cystoscopy and upper tract imaging (typically CT urogram and USS).

Investigation of glycosuria

Participants randomised to the glycosuria arm of cohort 2 will be investigated for the presence of glycosuria. Glycosuria was chosen as it is a validated urine test that can detect a meaningful disease (diabetes mellitus), whose outcomes are better detected at an early stage. If a single positive result is identified upon research nurse review of the glucose test

For peer review only - http://bmjopen.bmj.com/site/about/guidelines.xhtml

parameter, then the participant will be invited to attend an Early Detection Clinic within 4 – 6 weeks of assessment of their results. At the clinic, participants will undergo HbA1c blood testing. Those with elevated HbA1c (as defined by local reference ranges) will be referred to their GP for diabetic management. Those with a normal HbA1c (as defined by local reference ranges) will receive a letter explaining that their test was normal and will be advised to see their GP if they think they have diabetic symptoms. Their GP will also be informed of the test result. Participants in the glucosuria testing arm without glycosuria will receive a letter confirming their normal result and informed that their GP will also be notified of their results. Participants will be advised to seek a GP appointment if they develop diabetic symptoms.

Consent

Upon receipt of the postal urine self-testing kit, participants will be able to choose an action to take:

1. Consent to enter trial: Those wishing to take part in the trial will be asked to consent to participate by agreeing to consent statements in the mobile App or freephone system, before proceeding to complete the study questionnaire, and then self-testing with the test strips. Invitees will be prompted prior to providing results to have read the Participant Information Sheet.
2. Non-response: non-responders (i.e., do not consent or provide test results via the mobile App or freephone)

Following a positive urine self-test result for haematuria (Cohort 1) and haematuria/glucosuria (Cohort 2) participants will receive a telephone call from a study research nurse to explain their results and schedule an Early Detection Clinic appointment.

This will be followed by the mailout of a second PIS detailing what will happen at the Early Detection Clinic appointment. At the clinic, written informed consent will be obtained prior to any trial specific procedures (Supplemental Material). The CI will ensure that those with delegated responsibility to obtain informed consent are duly authorised, trained and competent according to the ethically approved protocol, principles of GCP and Declaration of Helsinki. The right of a participant to refuse participation without giving reasons will be respected.

For peer review only

For peer review only - http://bmjopen.bmj.com/site/about/guidelines.xhtml

Cohort 3 participants will receive a self-testing kit including a study invitation letter and PIS detailing what will happen at the 2ww appointment (figure 3). The invitation letter will be from the routine care team at the participating urology department. On presentation to the 2ww appointment, written informed consent will be obtained prior to any trial specific procedures. Translation via hospital interpreters is acceptable if required. The Principal Investigator (PI) will ensure that those with delegated responsibility to obtain informed consent are duly authorised, trained and competent according to the ethically approved protocol, principles of GCP and Declaration of Helsinki. The right of a participant to refuse participation without giving reasons will be respected. For Cohort 3, participation is mostly altruistic as the participants will already be in the NHS pathway and will have the gold-standard tests for detection of BCa.

Sample size(s)

A formal power calculation is not appropriate for this trial due to its feasibility related outcomes. The current planned sample size is to invite 2,000 participants in Cohort 1, (estimated 800 enrolled), invite 3,000 male participants in Cohort 2, (estimated to enrol and randomise 1,200) and invite 1,000 participants in Cohort 3 (estimated enrolment of 500).

Randomisation

Cohort 2 will be randomised between two arms where either the haematuria or glycosuria result will be revealed during the trial. Participants will be randomised via permuted blocks to ensure approximately equal numbers in the two arms. The randomisation lists will be generated by the trial statistician. The central nursing team will randomise participants prior to review of self-test results. Participants will not be given any details of the allocated group until they receive their initial results letter.

Primary Outcomes

This is a feasibility study and so there are multiple primary outcomes reflecting each stage of the study and what we need to understand for a larger phase 3 RCT:

1. Acceptability of urine self-testing and the Early Detection Clinics will be measured by;

    1.1. Proportion of invitees who report results for at least 1 test strip.

For peer review only - http://bmjopen.bmj.com/site/about/guidelines.xhtml

1.2. Proportion of participants who attend and consent for cytology and urinary tract ultrasound following detection of haematuria on self-testing.

1.3. Proportion of participants who attend and consent for HbA1c blood testing following the detection of glycosuria on self-testing.

2. Compliance with urine self-testing will be measured by;

2.1. The proportion of participants who report results for i) all 6 test strips, and ii) 3 or more test strips.

2.2. The number of test strips returned on average in those who return at least one test strip.

3. Prevalence of haematuria will be measured from the proportions of participants with ≥1 test strip positive for haematuria.

4. Prevalence of glycosuria will be measured from the proportions of participants with ≥1 test strip positive for glycosuria.

5. Haematuria clinic outcomes will be measured via:

5.1. The proportion of participants with a positive or abnormal cytology in those where a urine sample is collected.

5.2. The proportion of participants who have an abnormal ultrasound scan, who underwent scanning.

6. Glycosuria clinic outcomes will be measured via the proportions of those with an elevated HbA1c following collection of a blood sample.

7. Urinary symptoms will be measured via symptom scores from the urine symptoms questionnaire (as completed prior to self-testing).

8. BCa in the tested population will be measured via the proportions of BCa cases and corresponding stage in identified participants (excluding non-responders and withdrawals).

9. The accuracy of self-testing, cytology, and ultrasound scan will be measured via:

9.1. The sensitivity, specificity, positive predictive value, and negative predictive value of the following in men and women with cancer as identified by cystoscopy results at the 2 Week Wait appointment

9.1.1. Self-testing (App vs. freephone)

9.1.2. Cytology and ultrasound (separately and in combination) as a triage test in those with positive haematuria

9.2. The predictive value of symptoms in combination with self-testing:

For peer review only - http://bmjopen.bmj.com/site/about/guidelines.xhtml

9.2.1. Haematuria positive/symptoms positive

9.2.2. Haematuria negative/symptoms positive

9.2.3. Haematuria positive/symptoms negative

9.2.4. Haematuria negative/symptoms negative

10. Recruitment rates will be measured via the number of invitations sent out versus uptake.

Secondary and exploratory outcomes

Secondary outcomes include 1). self-test reporting via the mobile app or freephone, and this impact on sensitivity/specificity to detection of cancer, and 2). the acceptability of the reciprocal design (measured by the proportions of compliance/attendance/surveys at the end of the trial).

Exploratory outcomes include 1). Comparison of populations, demographics and cancer incidence in those who do/do not complete self-testing (comparing the characteristics where available including sex, age group, quintile of deprivation, smoking history). All participants that self-test will be flagged in NCRAS for cancer diagnoses at 1, 2 and 3 years following the end of the trial. 2). The development of an artificial intelligence (AI) platform for the assessment of urine samples for cytology analysis for the future study will be measured by the accuracy of the AI read versus the manual read at the end of the trial. 3). The potential harm of cystoscopy adverse effects, specifically urinary tract infections requiring medical treatment, will be measured by the proportions of participants that have a UTI requiring medical treatment following cystoscopy at the end of the trial. 4). Development of a health economics model [23] using the information from this feasibility study that could be used to develop a future community-based screening programme for bladder cancer will be measured at the end of the study by: 4.1. Resource usage in each arm (number of clinics used, blood tests, ultrasound tests, extra NHS tests) 4.2. Rate of cancers and other illnesses per arm. 5). The addition of other urinary contents (leukocytes, nitrate, protein) in relation to the risk prediction of bladder cancer will be measured by other positive test strip panels in addition to haematuria at the end of the trial.

Data collection

For peer review only

Self-test results and questionnaire data will be captured in the bespoke mobile app and freephone system. The questionnaire will collect information on diabetes (diagnosis and management), smoking status, ethnic group, LGBT identification, highest level of education, history of factory work as well as bladder symptoms (via a modified and unvalidated version of the Urinary Tract Infection Symptom Assessment questionnaire (UTISA) [25]). Cohort 1 and 2 data collected at Early Detection Clinics will be entered into a REDCap [26] database and will include height and weight, current occupation, previous longest occupation, concomitant medication, ultrasound results, urine cytology results, and HbA1c results. Data for Cohort 1 and 2 participants that are subsequently referred for a 2WW, and consenting Cohort 3 participants, will be obtained via medical records to include results of cystoscopy, ultrasound/CT, cytology, and cancer staging where applicable. At 14-30 days post cystoscopy, these participants will be contacted to collect information of any treated incidences of UTI following cystoscopy. Passive follow-up data (identification of cancer diagnosis and stage) will be collected through the National Cancer Registration and Analysis Service (NCRAS) at 1, 2, and 3 years after the end of the trial for all participants that have self-tested (excluding withdrawals). Summary data will also be collected through NCRAS, requested by geographic area, for a population comparable to Cohort 2.

Statistical Analysis

We will present estimates of percentages, with 95% Wilson confidence intervals, on a per cohort basis for the three trial endpoints that will allow assessment of feasibility and inform the subsequent more definitive trial. We will estimate acceptability (proportion of invitees consenting and returning a test strip), compliance (proportion of participants returning at least 3 or all 6 test strips), haematuria and glucosuria, proportion of positive outcomes for those referred to the Early Detection Clinic (positive cytology and/or USS for haematuria and HbA1c for glycosuria). We will assess the levels of BCa in the cohort identified by Early Detection clinic and at 3-years using National Disease Registration Service (NDRS, formerly NCRAS) and the accuracy of the haematuria test results including positive and negative predictive values (NPV and PPV) for cohorts 1 and 2 only. The urinary symptoms questionnaires will be summarised by cohort, by the proportions with each urinary symptom, and by overall urinary symptoms using mean, SD, median, minimum, maximum and quartiles. Missing values will be imputed as no symptom.

For peer review only - http://bmjopen.bmj.com/site/about/guidelines.xhtml

Economic evaluation

An early health economic model of haematuria screening for bladder cancer in high-risk areas will be developed, including epidemiology, natural history and how this is affected by screening [23]. The model design will be informed through analysis of other screening and diagnostic models in BCa. The model will be parameterised with the generated feasibility outcome data, supplemented by evidence from published literature. Bayesian calibration methods will be used for parameterisation of the disease natural history and screening model. The model will input to the design of and be refined using the subsequent trial outcomes.

Patient and public involvement (PPI)

A group of 9 PPI representatives were approached for initial review of the trial design, objectives, lay summary, and survey questions, and their responses have been incorporated in the trial design. PPI members have also reviewed the participant facing documentation and provided feedback. One-to-one interviews and discussions with PPI members were held to obtain specific feedback on aspects of how the study is presented to potential participants. A PPI lead is included in the Trial Steering Committee.

Trial organisation and administration

The trial is sponsored by the University of Sheffield and coordinated by the Cancer Research UK & King's College London Cancer Prevention Trials Unit (CPTU). The central research nursing team and central cytology laboratory is based at Sheffield Teaching Hospital NHS Foundation Trust.

Two trial oversight groups provide guidance and management for the three cohorts. The Trial Management Group meet at least monthly and will comprise the Chief Investigator, representatives from University of Sheffield, and CPTU who are responsible for day-to-day management of the trial and carrying out the work detailed in the trial protocol. The purpose of the group is to ensure the trial is on track to its project plan and budget, manage trial risks, issues, and mitigations and to escalate and report any major risks and issues to the Trial Steering Committee (TSC) as necessary.

For peer review only - http://bmjopen.bmj.com/site/about/guidelines.xhtml

The TSC will concentrate on progress of the trial, adherence to the protocol, participant welfare and to assess the impact and relevance of any new information of relevance to the research question. The TSC will make recommendations, through its Chair, to the TMG, Chief Investigator, trial sponsor, trial funders, CPTU and any other relevant party on all appropriate aspects of the trial. Most members of the TSC, including the Chair, will be independent of the trial, and will include lay/patient representation. Non-independent members will also be part of the TSC as observers. The final decision regarding whether the trial may continue is the responsibility of the TSC.

Ethics and Dissemination

A risk assessment and review of ethical considerations has been performed as part of the trial, including development of mitigation strategies to ensure the safe and successful delivery of the trial. Key risks and mitigations from this trial have been outlined below:

- Cancer screening is in part controversial due to the risks of finding and over treating indolent cancers. However, for BCa there is minimal morbidity in diagnosis (i.e., from the TURBT) and risk stratification is embedded in the treatment pathways. Many of these cancers are managed without radical treatment, and therefore there are few long-term side effects. Furthermore, autopsy studies [9] reveal few undiagnosed BCa's, suggesting that most tumours present clinically. Modelling of the disease natural history will aim to estimate overdiagnosis and will provide input to subsequent trial design to provide definitive evidence on these issues.
- Another risk is that participants may attempt to seek the same test outside of the trial or try to identify what the colour change on their urinary test strip represents. We have worked with PPI representatives to tailor trial communications. It is key that participants understand that participation in the trial should be seen as altruistic and there is currently no evidence to suggest that screening of this may be beneficial and indeed may cause harm. Liaison with local GPs throughout the pilot period will be key in determining if they observe any increase in men or women requesting urinalysis assessments.
- Variable practices in secondary care for the treatment of BCa (i.e., post study) is another risk. Treating clinicians may use a wide variety of different regimens) to manage screen detected cancers. As part of the planned future trial, we will apply for funding for an embedded cohort study of comparative effectiveness and health-related quality of life

For peer review only - http://bmjopen.bmj.com/site/about/guidelines.xhtml

(HRQOL) for these screen-detected cancers. Patients/clinicians will be able to choose from a selection of standard National Institute of Care Excellence (NICE) approved pathways. HRQOL and cost will be collected within these pathways.

To ensure widespread dissemination, we plan to circulate the trial results to multiple research outputs including but not limited to conference proceedings and peer-reviewed journal articles relating to the trial design, results of the pilot trial, final uptake of participants and a final main analysis of primary and secondary endpoints encompassing economic modelling aspects. All trials will be published in high-impact journals, accompanied by a press release. This will be covered in more detail in the trial publication policy. Dissemination of trial results will be designed and delivered after extensive collaboration with PPI groups.

Trial Status

The trial opened to recruitment on 3rd October 2022 and is in the active recruitment phase.

For peer review only

For peer review only - http://bmjopen.bmj.com/site/about/guidelines.xhtml

Acknowledgements:

The trial is led by Professor James Catto, sponsored by the University of Sheffield, and coordinated by the Cancer Research UK & King's College London Cancer Prevention Trials Unit (CPTU). The trial is supported by TestCard Ltd, InHealth, Sealed Envelope and iPlato Healthcare. The authors would like to thank Joanna Gambell and Harriet Strachan of the Kings College London Cancer Prevention Group for their expertise and assistance with the management, coordination and administration of the YORKSURe study. The authors would also like to thank Allen Knight of Action Bladder Cancer UK and Lydia Makaroff of World Bladder Cancer Patient Coalition/Fight Bladder Cancer for their review and guidance during the development of the study design. Study data were collected and managed using REDCap Research Electronic Data Capture, an electronic, web-based data capture tool hosted at the Cancer Prevention Group, King's College London.

Author contributions:

JWFC – concept, protocol development and manuscript (all)

BN – statistics and manuscript (statistics/trial design)

MG – protocol development, trial management and manuscipt (all)

AC– protocol development, trial management and construction of manuscipt (all)

MS – concept and protocol development

OM – health economic modelling

JCh – concept and health economic modelling

PS – concept, trial design, statistics, and manuscript (all)

MGC – concept, protocol development and manuscipt (all)

Ethics Approvals:

The study has received the following approvals: London – Riverside Research Ethics Committee (22/LO/0018), Health Research Authority Confidentiality Advisory Group (20/CAG/0009).

Participant Consent for Publication:

Not required. No identifiable personal data will be used in publications.

For peer review only

Data Availability:

All information related to participants will be kept confidential and managed in accordance with UK General Data Protection Regulation (GDPR), Data Protection Act (2018), NHS Caldicott Principles, UK Policy Framework for Health and Social Care Research (2017), and the conditions of Research Ethics Committee Approval. Upon reasonable requests to the study team, only deidentified participant data will be available after publication of the study outcomes. Use and projects need approval by the Trial Steering Committee. Data will be shared via secure NHS email or a secure data sharing platform. Robust data sharing agreements will be put in place with all collaborating organisations as necessary to ensure the confidentiality and appropriate data handling. No identifiable personal data will be shared with organisations or individuals outside of these collaborating organisations.

Competing interests:

JWFC has received reimbursement for consultancy from Astra Zeneca, BMS, Ipsen, Janssen and Roche, speaker fees from BMS, Ipsen, MSD, Nucleix and Roche, honoraria for membership of advisory boards from Astra Zeneca, Ferring, Roche and Janssen, and research funding from Roche. PS is a paid member of the Scientific Advisory Board of GRAIL and the medical advisory board of NSV. The remaining authors declare no potential conflicts of interest.

Funding:

This work was funded by Yorkshire Cancer Research (Grant number RA/2019/R1/004: S431 Early detection of bladder cancer in Yorkshire: Feasibility assessments for implementing a targeted study in populations with high disease specific mortality risk). JWFC is funded by an NIHR Research Professorship (NIHR300047). MGC is funded by the NIHR (senior lecturer) and is in receipt of a RCSEng Hunterian Professorship (Royal College of Surgeons of England, 2023). BN and MS are funded by a Cancer Research UK grant led by PS (C8162/A25356). The NIHR have funded the service support costs and Part B research costs associated with the study. TestCard Ltd have provided an in-kind donation for the develop of the YORKSURe Mobile App. The funders had no role in the design, analysis or collection of the data; in writing the manuscript; or in the decision to submit the manuscript for publication.

For peer review only

References

[1] Cumberbatch MGK, Jubber I, Black PC, Esperto F, Figueroa JD, Kamat AM, et al. Epidemiology of Bladder Cancer: A Systematic Review and Contemporary Update of Risk Factors in 2018. Eur Urol. 2018;74:784-95.

[2] Catto JWF, Mandrik O, Quayle LA, Hussain SA, McGrath J, Cresswell J, et al. Diagnosis, treatment and survival from bladder, upper urinary tract, and urethral cancers: real-world findings from NHS England between 2013 and 2019. BJU Int. 2023;131:734-44.

[3] Catto JWF, Khetrapal P, Ricciardi F, Ambler G, Williams NR, Al-Hammouri T, et al. Effect of Robot-Assisted Radical Cystectomy With Intracorporeal Urinary Diversion vs Open Radical Cystectomy on 90-Day Morbidity and Mortality Among Patients With Bladder Cancer: A Randomized Clinical Trial. JAMA. 2022;327:2092-103.

[4] Catto JWF, Gordon K, Collinson M, Poad H, Twiddy M, Johnson M, et al. Radical Cystectomy Against Intravesical BCG for High-Risk High-Grade Nonmuscle Invasive Bladder Cancer: Results From the Randomized Controlled BRAVO-Feasibility Study. J Clin Oncol. 2021;39:202-14.

[5] Jubber I, Ong S, Bukavina L, Black PC, Comperat E, Kamat AM, et al. Epidemiology of Bladder Cancer in 2023: A Systematic Review of Risk Factors. Eur Urol. 2023 Aug;84(2):176-190.

[6] Cumberbatch MG, Cox A, Teare D, Catto JW. Contemporary Occupational Carcinogen Exposure and Bladder Cancer: A Systematic Review and Meta-analysis. JAMA Oncol. 2015;1:1282-90.

[7] Cumberbatch MG, Rota M, Catto JW, La Vecchia C. The Role of Tobacco Smoke in Bladder and Kidney Carcinogenesis: A Comparison of Exposures and Meta-analysis of Incidence and Mortality Risks. Eur Urol. 2016;70:458-66.

[8] NHS Digital. Cancer incidence and mortality 2021 [National Disease Registration Service: Comparisons by NHS integrated care boards]. Available from: https://www.cancerdata.nhs.uk/incidence_and_mortality accessed 31st January 2023.

[9] Catto JWF, Rogers Z, Downing A, Mason SJ, Jubber I, Bottomley S, et al. Lifestyle Factors in Patients with Bladder Cancer: A Contemporary Picture of Tobacco Smoking, Electronic Cigarette Use, Body Mass Index, and Levels of Physical Activity. Eur Urol Focus. 2023 Apr 18;S2405-4569(23)00101-3. doi: 10.1016/j.euf.2023.04.003..

[10] Reed O, Jubber I, Griffin J, Noon AP, Goodwin L, Hussain S, et al. Occupational bladder cancer: A cross section survey of previous employments, tasks and exposures matched to cancer phenotypes. PLoS One. 2020;15:e0239338.

[11] Eylert MF, Hounsome, L. S.; Persad, R.; Bahl, A.; Jefferies, E.; Verne, J.; Mostafid, H. Falling bladder cancer incidence from 1990 to 2009 is not producing universal mortality improvements. Journal of Clinical Urology. 2014;7:90-8.

For peer review only - http://bmjopen.bmj.com/site/about/guidelines.xhtml

[12] Lyratzopoulos G, Saunders CL, Abel GA, McPhail S, Neal RD, Wardle J, et al. The relative length of the patient and the primary care interval in patients with 28 common and rarer cancers. Br J Cancer. 2015;112 Suppl 1:S35-40.

[13] Jubber I, Shariat SF, Conroy S, Tan WS, Gordon PC, Lotan Y, et al. Non-visible haematuria for the Detection of Bladder, Upper Tract, and Kidney Cancer: An Updated Systematic Review and Meta-analysis. Eur Urol. 2020;77:583-98.

[14] Larre S, Catto JW, Cookson MS, Messing EM, Shariat SF, Soloway MS, et al. Screening for bladder cancer: rationale, limitations, whom to target, and perspectives. Eur Urol. 2013;63:1049-58.

[15] Elliott J, Rotterud R, Maddox-Smith A, Bagshaw K, Cirefice LBF, Knight A, et al. The bladder cancer patient survey: Global perspectives on awareness and treatment of bladder cancer. Journal of Cancer Policy. 2019;22:100207.

[16] Makaroff LE, Filicevas A, Boldon S, Hensley P, Black PC, Chisolm S, et al. Patient and Carer Experiences with Bladder Cancer: Results from a Global Survey in 45 Countries. Eur Urol. 2023.

[17] Britton JP, Dowell AC, Whelan P. Dipstick haematuria and bladder cancer in men over 60: results of a community study. BMJ. 1989;299:1010-2.

[18] Messing EM, Young TB, Hunt VB, Gilchrist KW, Newton MA, Bram LL, et al. Comparison of bladder cancer outcome in men undergoing hematuria home screening versus those with standard clinical presentations. Urology. 1995;45:387-96; discussion 96-7.

[19] Mayfield MP, Whelan P. Bladder tumours detected on screening: results at 7 years. Br J Urol. 1998;82:825-8.

[20] Thomas F, Rosario DJ, Rubin N, Goepel JR, Abbod MF, Catto JW. The long-term outcome of treated high-risk nonmuscle-invasive bladder cancer: time to change treatment paradigm? Cancer. 2012;118:5525-34.

[21] Messing EM, Young TB, Hunt VB, Newton MA, Bram LL, Vaillancourt A, et al. Hematuria home screening: repeat testing results. J Urol. 1995;154:57-61.

[22] Zlotta AR, Roumeguere T, Kuk C, Alkhateeb S, Rorive S, Lemy A, et al. Select screening in a specific high-risk population of patients suggests a stage migration toward detection of non-muscle-invasive bladder cancer. Eur Urol. 2011;59:1026-31.

[23] Mandrik O, Hahn AI, Catto JWF, Zauber AG, Cumberbatch M, Chilcott J. Critical Appraisal of Decision Models Used for the Economic Evaluation of Bladder Cancer Screening and Diagnosis: A Systematic Review. PharmacoEconomics. 2023 Jun;41(6):633-650.

[24] Crosbie PA, Gabe R, Simmonds I, Kennedy M, Rogerson S, Ahmed N, et al. Yorkshire Lung Screening Trial (YLST): protocol for a randomised controlled trial to evaluate invitation to community-based low-dose CT screening for lung cancer versus usual care in a targeted population at risk. BMJ Open. 2020;10:e037075.

For peer review only

For peer review only - http://bmjopen.bmj.com/site/about/guidelines.xhtml

[25] Clayson D, Wild D, Doll H, Keating K, Gondek K. Validation of a patient-administered questionnaire to measure the severity and bothersomeness of lower urinary tract symptoms in uncomplicated urinary tract infection (UTI): the UTI Symptom Assessment questionnaire. BJU Int. 2005;96:350-9.

[26] Harris PA, Taylor R, Thielke R, Payne J, Gonzalez N, Conde JG. Research electronic data capture (REDCap)--a metadata-driven methodology and workflow process for providing translational research informatics support. J Biomed Inform. 2009;42:377-81.

For peer review only

Figures Legends

Figure 1. Recruitment and flow within Cohort 1 within the YORKSURe study: Male and Female participants will be solely recruited from the Yorkshire Lung Screening Trial (YLST).

Figure 2. Recruitment and flow within Cohort 2 of the YORKSURe study: Male participants will be invited from populations at risk of high-mortality from bladder cancer.

Figure 3. Participant flow within Cohort 3 of the YORKSURe study: Male and female patients undergoing investigation within the NHS for haematuria (according to the 2 week wait criteria within the NICE guidelines) will be invited to participate.

For peer review only

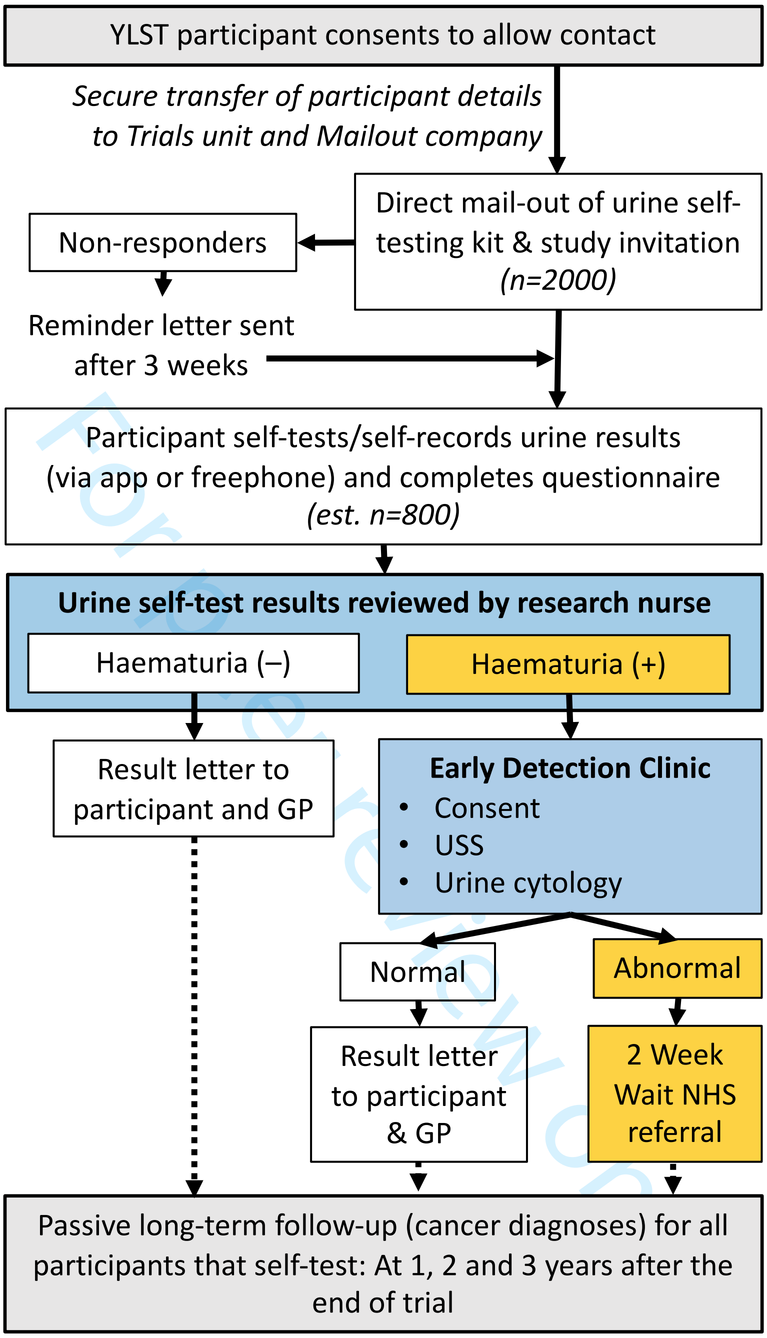

For peer review only - http://bmjopen.bmj.com/site/about/guidelines.xhtml

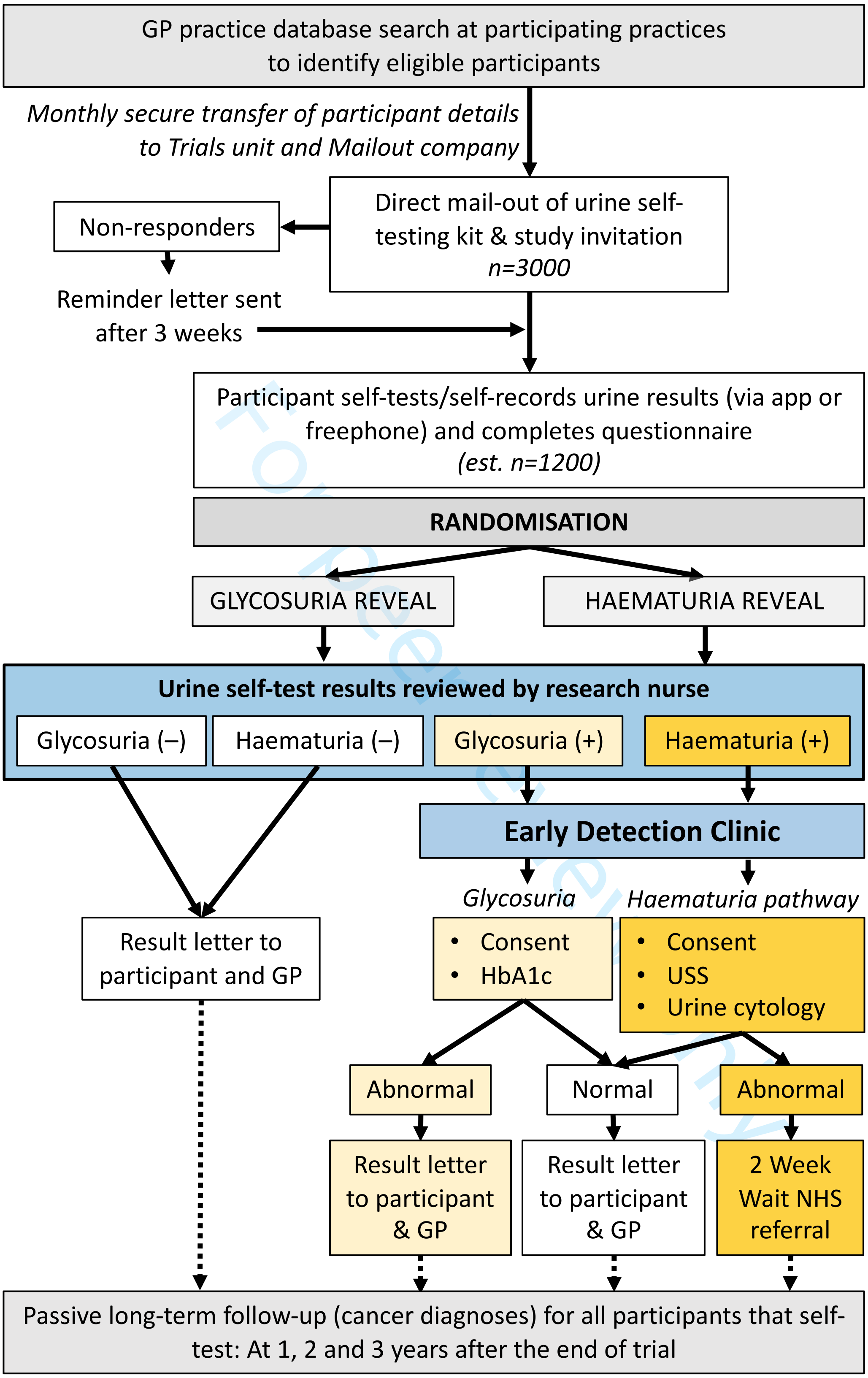
GP practice database search at participating practices to identify eligible participants
Monthly secure transfer of participant details to Trials unit and Mailout company
Direct mail-out of urine self-testing kit & study invitation n=3000
Non-responders
Reminder letter sent after 3 weeks
Participant self-tests/self-records urine results (via app or freephone) and completes questionnaire (est. n=1200)
RANDOMISATION
GLYCOSURIA REVEAL
HAEMATURIA REVEAL
Urine self-test results reviewed by research nurse
Glycosuria (–)
Haematuria (–)
Glycosuria (+)
Haematuria (+)
Early Detection Clinic
Glycosuria
Haematuria pathway
Result letter to participant and GP
Consent
HbA1c
Consent
USS
Urine cytology
Abnormal
Normal
Abnormal
Result letter to participant & GP
Result letter to participant & GP
2 Week Wait NHS referral
Passive long-term follow-up (cancer diagnoses) for all participants that self-test: At 1, 2 and 3 years after the end of trial

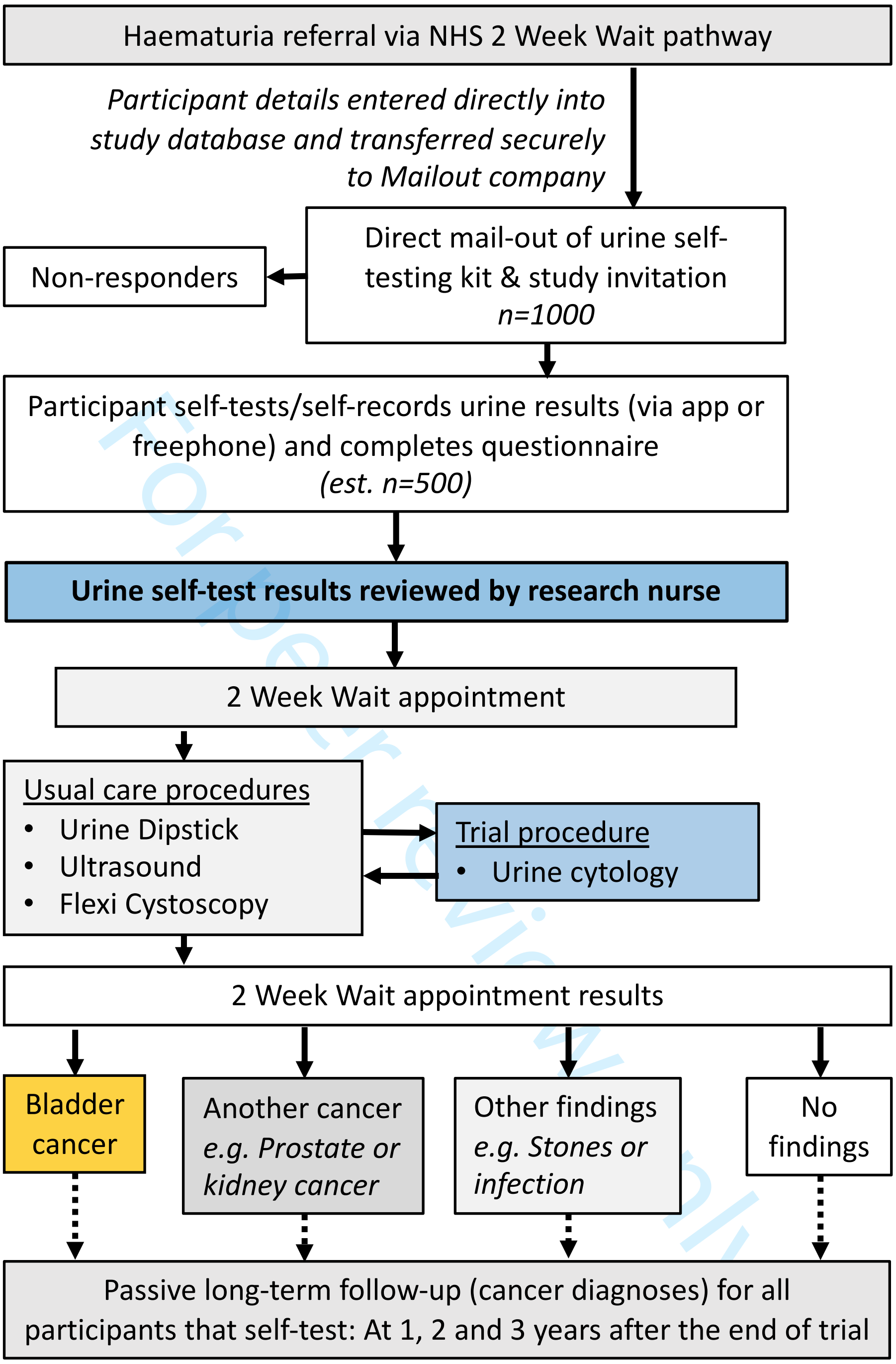

For peer review only - http://bmjopen.bmj.com/site/about/guidelines.xhtml

Title: Yorkshire Screening of Urine trial (YORKSURe)

IRAS ID: 302276

Study ID:

INFORMED CONSENT FORM

Name of Researcher: Professor James Catto

		Please initial box
1	I confirm that I have read and understood the participant information sheet (dated..................version..........) for the above study. I have had the opportunity to consider the information, ask questions and have had these answered to my satisfaction.	☐
2	I understand that taking part is voluntary and that I am free to withdraw at any time without giving any reason, and without my medical care or legal rights being affected. I understand that samples and data collected prior to my withdrawal will continue to be used for the purposes of the study.	☐
3	I understand that relevant sections of my medical notes and data collected during the study, may be looked at by individuals from King's College London, University of Sheffield, or from regulatory authorities or from the NHS Trust, where it is relevant to my taking part in this research. Access to data by the organisations above may be given for monitoring and/or audit of the study to ensure that the research is complying with applicable research regulations. I give permission for these individuals to have access to my records and data.	☐
4	I understand that my personal data will be shared solely between members of the research team and participating study sites as part of the data analysis required for this study. As detailed in the participant information sheet, I am aware that employees of third-party providers contracted by King's College London may require access to my data to fulfil their role as a third-party service provider, but my records and information will be kept strictly confidential.	☐

For peer review only - http://bmjopen.bmj.com/site/about/guidelines.xhtml

**5**	I understand that the information collected about me will be used to support other research in the future and may be shared anonymously with other researchers or organisations which may include those in the commercial sector, here or abroad.	☐
**6**	**Applicable if providing a urine sample only:** I understand that my anonymised urine sample (slide) may be used in future research, here or abroad, which has ethics approval. I understand this research may involve commercial organisations.	☐ Yes ☐ N/A
**7**	I agree to my General Practitioner being informed that I am taking part in the study and that my test results will be shared with them.	☐
**8**	I agree to take part in the above study.	☐

Name of participant	Date	Signature
Name of Person seeking consent	Date	Signature

For peer review only

For peer review only - http://bmjopen.bmj.com/site/about/guidelines.xhtml

SPIRIT Checklist: Protocol for the YORKSURe prospective multi-stage study testing the feasibility for early detection of bladder cancer in populations with high disease specific mortality risk

Section/item	Item No	Description	
**Administrative information**			
Title	1	Descriptive title identifying the study design, population, interventions, and, if applicable, trial acronym	Page 1
Trial registration	2a	Trial identifier and registry name. If not yet registered, name of intended registry	Page 2
	2b	All items from the World Health Organization Trial Registration Data Set	Throughout the manuscript
Protocol version	3	Date and version identifier	Protocol Vsn. 5.0 date 16/1/2023, as detailed on page 5.
Funding	4	Sources and types of financial, material, and other support	Detailed on Page 17
Roles and responsibilities	5a	Names, affiliations, and roles of protocol contributors	Page 1
	5b	Name and contact information for the trial sponsor	Page 13
	5c	Role of study sponsor and funders, if any, in study design; collection, management, analysis, and interpretation of data; writing of the report; and the decision to submit the report for publication, including whether they will have ultimate authority over any of these activities	Page 17
	5d	Composition, roles, and responsibilities of the coordinating centre, steering committee, endpoint adjudication committee, data management team, and other individuals or groups overseeing the trial, if applicable (see Item 21a for data monitoring committee)	Pages 14
**Introduction**			
Background and rationale	6a	Description of research question and justification for undertaking the trial, including summary of relevant studies (published and unpublished) examining benefits and harms for each intervention	Pages 4-5
	6b	Explanation for choice of comparators	Page 8
Objectives	7	Specific objectives or hypotheses	Page 5
Trial design	8	Description of trial design including type of trial (eg, parallel group, crossover, factorial, single group), allocation ratio, and framework (eg, superiority, equivalence, noninferiority, exploratory)	Pages 6-7 and figures 1-3
**Methods: Participants, interventions, and outcomes**			
Study setting	9	Description of study settings (eg, community clinic, academic hospital) and list of countries where data will be collected. Reference to where list of study sites can be obtained	Page 6
Eligibility criteria	10	Inclusion and exclusion criteria for participants. If applicable, eligibility criteria for study centres and individuals who will perform the interventions (eg, surgeons, psychotherapists)	Page 6-7

For peer review only

Interventions	11a	Interventions for each group with sufficient detail to allow replication, including how and when they will be administered	Pages 6-7 and figures 1-3
	11b	Criteria for discontinuing or modifying allocated interventions for a given trial participant (eg, drug dose change in response to harms, participant request, or improving/worsening disease)	NA
	11c	Strategies to improve adherence to intervention protocols, and any procedures for monitoring adherence (eg, drug tablet return, laboratory tests)	NA
	11d	Relevant concomitant care and interventions that are permitted or prohibited during the trial	NA
Outcomes	12	Primary, secondary, and other outcomes, including the specific measurement variable (eg, systolic blood pressure), analysis metric (eg, change from baseline, final value, time to event), method of aggregation (eg, median, proportion), and time point for each outcome. Explanation of the clinical relevance of chosen efficacy and harm outcomes is strongly recommended	Pages 10-12
Participant timeline	13	Time schedule of enrolment, interventions (including any run-ins and washouts), assessments, and visits for participants. A schematic diagram is highly recommended (see Figure)	Page 6 and figures 1-3
Sample size	14	Estimated number of participants needed to achieve study objectives and how it was determined, including clinical and statistical assumptions supporting any sample size calculations	Figures 1-3 and page 10
Recruitment	15	Strategies for achieving adequate participant enrolment to reach target sample size	Pages 7-10
**Methods: Assignment of interventions (for controlled trials)**			
Allocation Sequence generation	16a	Method of generating the allocation sequence (eg, computer-generated random numbers), and list of any factors for stratification. To reduce predictability of a random sequence, details of any planned restriction (eg, blocking) should be provided in a separate document that is unavailable to those who enrol participants or assign interventions	Page 10
Allocation concealment mechanism	16b	Mechanism of implementing the allocation sequence (eg, central telephone; sequentially numbered, opaque, sealed envelopes), describing any steps to conceal the sequence until interventions are assigned	Page 10
Implementation	16c	Who will generate the allocation sequence, who will enrol participants, and who will assign participants to interventions	Page 10
Blinding (masking)	17a	Who will be blinded after assignment to interventions (eg, trial participants, care providers, outcome assessors, data analysts), and how	Page 10
	17b	If blinded, circumstances under which unblinding is permissible, and procedure for revealing a participant's allocated intervention during the trial	No unblinding is anticipated
**Methods: Data collection, management, and analysis**			

Data collection methods	18a	Plans for assessment and collection of outcome, baseline, and other trial data, including any related processes to promote data quality (eg, duplicate measurements, training of assessors) and a description of study instruments (eg, questionnaires, laboratory tests) along with their reliability and validity, if known. Reference to where data collection forms can be found, if not in the protocol	Page 12
	18b	Plans to promote participant retention and complete follow-up, including list of any outcome data to be collected for participants who discontinue or deviate from intervention protocols	Rapid completion of trial procedures.
Data management	19	Plans for data entry, coding, security, and storage, including any related processes to promote data quality (eg, double data entry; range checks for data values). Reference to where details of data management procedures can be found, if not in the protocol	Page 12 and Page 18 (under data availability)
Statistical methods	20a	Statistical methods for analysing primary and secondary outcomes. Reference to where other details of the statistical analysis plan can be found, if not in the protocol	Page 13
	20b	Methods for any additional analyses (eg, subgroup and adjusted analyses)	Page 12
	20c	Definition of analysis population relating to protocol non-adherence (eg, as randomised analysis), and any statistical methods to handle missing data (eg, multiple imputation)	Page 6
**Methods: Monitoring**			
Data monitoring	21a	Composition of data monitoring committee (DMC); summary of its role and reporting structure; statement of whether it is independent from the sponsor and competing interests; and reference to where further details about its charter can be found, if not in the protocol. Alternatively, an explanation of why a DMC is not needed	There is no DMC for this feasibility study. This was decised using advice from the EMEA and FDA. Key factors for the decision were the short term nature of the outcomes, non-drug design and that the test performances are well known for not harming patients.
	21b	Description of any interim analyses and stopping guidelines, including who will have access to these interim results and make the final decision to terminate the trial	Nil
Harms	22	Plans for collecting, assessing, reporting, and managing solicited and spontaneously reported adverse events and other unintended effects of trial interventions or trial conduct	Pages 12-13
Auditing	23	Frequency and procedures for auditing trial conduct, if any, and whether the process will be independent from investigators and the sponsor	Pages 12-13
**Ethics and dissemination**			
Research ethics approval	24	Plans for seeking research ethics committee/institutional review board (REC/IRB) approval	Page 14

Protocol amendments	25	Plans for communicating important protocol modifications (eg, changes to eligibility criteria, outcomes, analyses) to relevant parties (eg, investigators, REC/IRBs, trial participants, trial registries, journals, regulators)	NA
Consent or assent	26a	Who will obtain informed consent or assent from potential trial participants or authorised surrogates, and how (see Item 32)	Page 8
	26b	Additional consent provisions for collection and use of participant data and biological specimens in ancillary studies, if	NA
Confidentiality	27	How personal information about potential and enrolled participants will be collected, shared, and maintained in order to protect confidentiality before, during, and after the trial	Page 18 (under data availability)
Declaration of interests	28	Financial and other competing interests for principal investigators for the overall trial and each study site	Page 18
Access to data	29	Statement of who will have access to the final trial dataset, and disclosure of contractual agreements that limit such access for investigators	Page 18 under data availability
Ancillary and post-trial care	30	Provisions, if any, for ancillary and post-trial care, and for compensation to those who suffer harm from trial participation	NA
Dissemination policy	31a	Plans for investigators and sponsor to communicate trial results to participants, healthcare professionals, the public, and other relevant groups (eg, via publication, reporting in results databases, or other data sharing arrangements), including any publication restrictions	Page 15-16
	31b	Authorship eligibility guidelines and any intended use of professional writers	Page 17 - Author contributions
	31c	Plans, if any, for granting public access to the full protocol, participant-level dataset, and statistical code	Page 18 - Data Availability
**Appendices**			
Informed consent materials	32	Model consent form and other related documentation given to participants and authorised surrogates	Supplementary figure 1
Biological specimens	33	Plans for collection, laboratory evaluation, and storage of biological specimens for genetic or molecular analysis in the current trial and for future use in ancillary studies, if applicable	NA

For peer review only
