# Supplementary material for: Protocol for the YORKSURe prospective multistage study testing the feasibility for early detection of bladder cancer in populations with high disease-specific mortality risk
Source: BMJ Open. 2023 Sep 7;13(9):e076612. doi: 10.1136/bmjopen-2023-076612 (PMC10496676; doi:10.1136/bmjopen-2023-076612)
Supplement: Reviewer comments [file bmjopen-2023-076612.reviewer_comments.pdf]

PEER REVIEW HISTORY

BMJ Open publishes all reviews undertaken for accepted manuscripts. Reviewers are asked to complete a checklist review form (**http://bmjopen.bmj.com/site/about/resources/checklist.pdf**) and are provided with free text boxes to elaborate on their assessment. These free text comments are reproduced below.

ARTICLE DETAILS

**TITLE (PROVISIONAL)**	Protocol for the YORKSURe prospective multi-stage study testing the feasibility for early detection of bladder cancer in populations with high disease specific mortality risk
**AUTHORS**	Catto, James; North, Bernard; Goff, Megan; Carter, Abigail; Sleeth, Michelle; Mandrik, Olena; Chilcott, Jim; Sasieni, Peter; Cumberbatch, Marcus

This article was not externally reviewed at BMJ Open. Protocols that have been independently assessed prior to submission to BMJ Open are usually fast-tracked to publication on the grounds that further substantial changes will not be possible. This independent assessment will usually be external, independent review for both a substantial grant award from a non-commercial or government funder and ethics approval.
